# The extracellular vesicle of gut microbial *Paenalcaligenes hominis* is a risk factor for vagus nerve-mediated cognitive impairment

**DOI:** 10.1186/s40168-020-00881-2

**Published:** 2020-07-15

**Authors:** Kyung-Eon Lee, Jeon-Kyung Kim, Sang-Kap Han, Dong Yun Lee, Hae-Ji Lee, Sung-Vin Yim, Dong-Hyun Kim

**Affiliations:** 1 Neurobiota Research Center, Department of Life and Nanopharmaceutical Sciences, College of Pharmacy, Kyung Hee University, 26, Kyungheedae-ro, Dongdaemun-gu, Seoul, 02447 South Korea; 2 Department of Clinical Pharmacology and Therapeutics, College of Medicine, Kyung Hee University, Seoul, 02447 South Korea

**Keywords:** *Paenalcaligenes hominis*, Extracellular vesicle, Lipopolysaccharide, Cognitive decline, Colitis

## Abstract

**Background:**

In a pilot study, we found that feces transplantation from elderly individuals to mice significantly caused cognitive impairment. *Paenalcaligenes hominis* and *Escherichia coli* are increasingly detected in the feces of elderly adults and aged mice. Therefore, we isolated *Paenalcaligenes hominis* and *Escherichia coli* from the feces of elderly individuals and aged mice and examined their effects on the occurrence of age-related degenerative cognitive impairment and colonic inflammation in mice.

**Results:**

The transplantation of feces collected from elderly people and aged mice caused significantly more severe cognitive impairment in transplanted young mice than those from young adults and mice. Oral gavage of *Paenalcaligenes hominis* caused strong cognitive impairment and colitis in specific pathogen-free (SPF) and germ-free mice. *Escherichia coli* also induced cognitive impairment and colitis in SPF mice. Oral gavage of *Paenalcaligenes hominis*, its extracellular vesicles (EVs), and/or lipopolysaccharide caused cognitive impairment and colitis in mice. However, celiac vagotomy significantly inhibited the occurrence of cognitive impairment, but not colitis, in mice exposed to *Paenalcaligenes hominis* or its EVs, whereas its lipopolysaccharide or *Escherichia coli* had no such effects. Vagotomy also inhibited the infiltration of EVs into the hippocampus.

**Conclusions:**

*Paenalcaligenes hominis*, particularly its EVs, can cause cognitive function-impaired disorders, such as Alzheimer’s disease, and its EVs may penetrate the brain through the blood as well as the vagus nerve.

Video Abstract

## Background

Alzheimer’s disease (AD), a multifactorial, complex, and neurodegenerative disorder, is the most common cause of dementia in the elderly [1, 2]. The major risk factor for AD is age, followed by metabolic disorders, chronic inflammation, and pathogen infection [3, 4]. Aging chronically stimulates the secretion of senescence-linked mediators, including pro-inflammatory cytokines, such as tumor necrosis factor (TNF)-α, and nuclear factor (NF)-κB, in the brain, while the brain-derived neurotrophic factor (BDNF) expression is downregulated [5–7]. Moreover, aging also increases lipopolysaccharide (LPS) production in the gut microbiota, accelerating cognitive impairment [8, 9]. The gastrointestinal microbiota of aged mice also accelerates inflammation more potently than that of young mice [8].

The gastrointestinal microbiota, comprising beneficial and pathogenic microbes such as *Bifidobacteria* and *Escherichia*, modulates the immune responses of hosts [10, 11]. However, the gastrointestinal microbiota is affected by a variety of endogenous and exogenous factors such as diet, drugs, hormones, and aging [12, 13]. Mitsuoka reported that *Bifidobacterium* populations were smaller in elderly adults than in young adults, whereas those of *Enterococcus*, *Lactobacillus*, and *Clostridium perfringens* were larger [14]. *Lactobacillus* and *Bifidobacterium* populations decline in elderly adults compared with their levels in young adults, whereas the abundance of *Eubacterium* and *Bacteroides* populations are not affected by aging [15–17]. In addition, the population of the anti-inflammatory microbe *Faecalibacterium prausnitzii*, belonging to *Clostridium* cluster IV, is remarkably small in Italian elderly adults and centenarian populations [18, 19], whereas there is an increased abundance of inflammatory gastrointestinal bacteria, such as Proteobacteria, in elderly and aged mice [20–22]. In addition, *Paenalcaligenes hominis*, a member of Proteobacteria, was frequently detected in the elderly, but not in children and young adults [23]. *Paenalcaligenes hominis* was also isolated from an elderly paraplegic patient with neurogenic bladder in Sweden [24]. Lee et al. reported that Proteobacteria, particularly *Paenalcaligenes hominis*, was frequently highly detected in aged mice, but not in young mice [25]. The intrarectal injection of 2,4,6-trinitrobenzenesulfonic acid (TNBS) simultaneously causes colonic inflammation and cognitive decline in mice and shifted the gastrointestinal microbiota composition, particularly increasing the Proteobacteria population [26]. The oral administration of TNBS-inducible *Escherichia coli* also causes memory impairment in mice [26]. Moreover, the excessive production of gastrointestinal bacterial byproducts such as LPS and kynurenine due to gut dysbiosis causes gastrointestinal and systemic inflammation, leading to inflammatory bowel disease and neuroinflammation [26–28]. These findings suggest that alteration of the gastrointestinal microbiota composition following microbial infection is intimately connected with the occurrence of cognitive decline, including AD, in elderly humans and aged mice.

Therefore, to understand the etiological commensal gastrointestinal bacteria responsible for cognitive decline in elderly adults and aged mice, we transplanted the feces of elderly people or aged mice, which produced significantly more colonies when grown on Enterobacteriaceae-selective deoxycholate hydrogen sulfide lactose (DHL) agar plates than those from young adults or mice, respectively (Supplement Figure S1), into young mice. The transplantation of feces collected from elderly people and aged mice caused significantly more severe cognitive impairment in transplanted young mice than those from young adults and mice, respectively. Thereafter, we isolated the gastrointestinal bacteria *Paenalcaligenes hominis* and *Escherichia coli*, which were excessively detectable in elderly people and aged mice [21, 23, 29, 30], and examined their effects on the occurrence of age-related degenerative cognitive impairment and colonic inflammation in mice.

## Results

### Transplantation of feces collected from elderly people or aged mice into young mice induced cognitive impairment

**Fig. 1:**
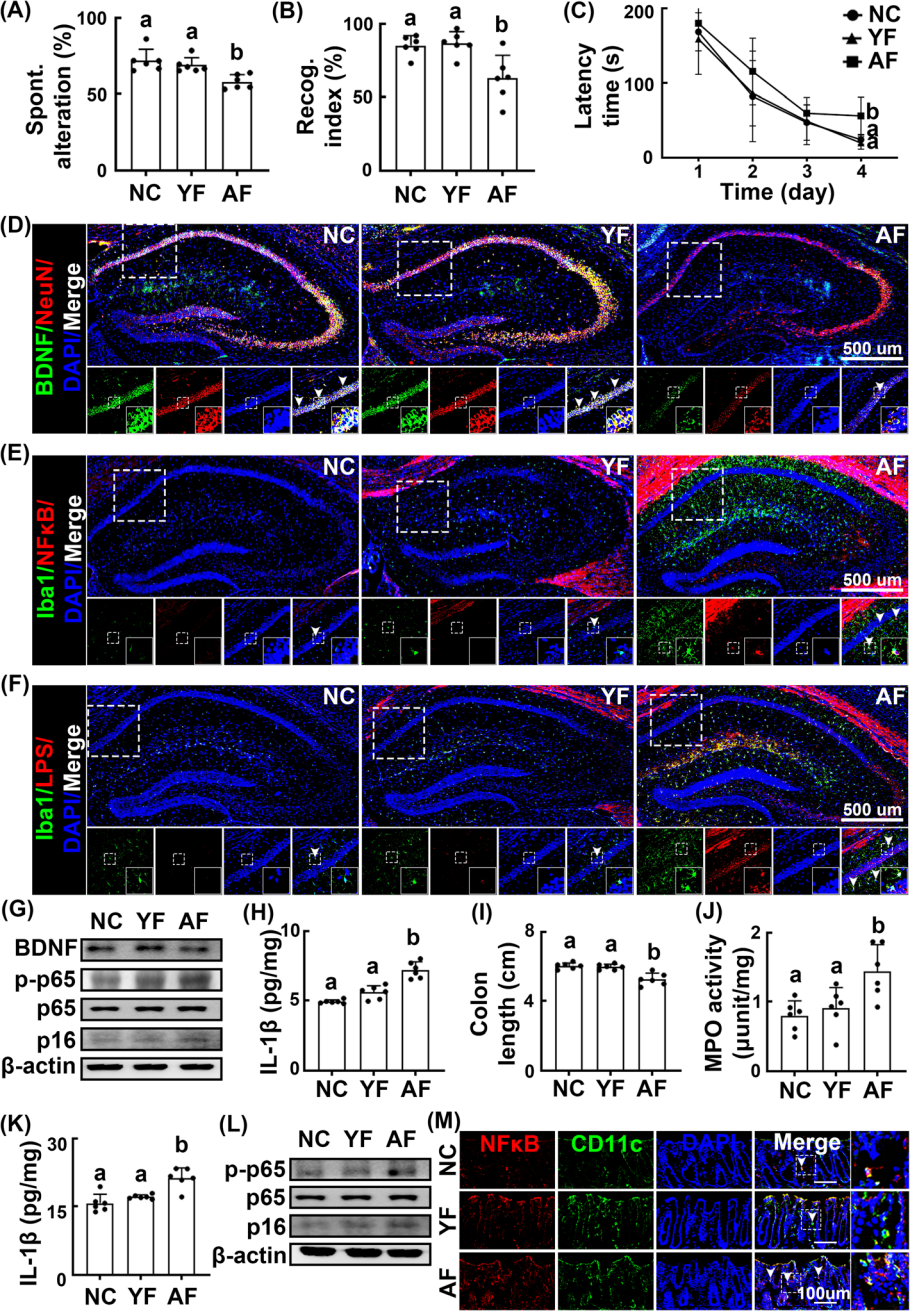
Effects of young adult and elderly feces on the occurrence of cognitive impairment and colitis in the transplanted mice. Effects on the occurrence of cognitive impairment in Y-maze (**A**), NOR (**B**), and Barnes maze tasks (**C**). Effects on the BDNF^+^/NeuN^+^ (**D**), NF-κB^+^/Iba1^+^ (**E**), and LPS^+^/Iba1^+^ cell populations (**F**) in the hippocampus. **G** Effects on the BDNF expression and NF-κB activation in the hippocampus. **H** Effects on the IL-1β expression in the hippocampus, assessed by ELISA. Effects on the colon length (**I**), myeloperoxidase (MPO) activity (**J**), IL-1β expression (**K**), NF-κB activation, p16 expression (**L**), and NF-κB^+^/CD11c^+^ cell population (**M**) in the colon. Fecal transplantations of young adults (YF) and elderly people (AF) were orally gavaged once a day for 5 days. Control mice (NC) were treated with vehicle (saline) instead of fecal suspension. Data values were indicated as mean ± SD (*n* = 6). Means with the same letters are not significantly different (*p* < 0.05). **A**, **B**, **C**, **I** Kruskal-Wallis test with Dunn’s post hoc test for non-parametric analysis. **H**, **J**, **K** One-way ANOVA with post hoc Bonferroni’s multiple comparisons test

To examine whether age-associated gastrointestinal microbes were involved in the impairment of cognitive function, we transplanted the feces of elderly people or aged mice into young mice and examined their effects on cognitive function in the Y-maze, novel object recognition (NOR), and Barnes maze tasks (Fig. 1A–C, Supplement Figure S2A–C). Fecal transplantation significantly induced cognitive impairment in the transplanted young mice. Fecal transplantation also decreased the BDNF expression and BDNF^+^/NeuN^+^ cell counts in the hippocampus, whereas the NF-κB^+^/Iba1^+^ and LPS^+^/Iba1^+^cell populations, NF-κB activation, and p16 and interleukin (IL)-1β expression increased (Fig. 1D–H, Supplement Figure S2D–H, Supplement Figure S3A–D). Fecal transplantation induced colitis in young mice, as indicated by colon shortening and an increase in myeloperoxidase activity, NF-κB activation, p16 and IL-1β expression, and NF-κB^+^/CD11c^+^ cell counts in the colon (Fig. 1H –M, Supplement Figure S2I–K, Supplement Figure S3E, F).

### *Paenalcaligenes hominis* and *Escherichia coli* caused severe cognitive decline and colitis in mice

The transplantation of feces from elderly people or aged mice, uniquely containing larger populations of *Paenalcaligenes hominis* and *Escherichia coli* than young adults or young mice (Supplement Figure S1), caused cognitive impairment and colitis in transplanted young mice. Therefore, we isolated *Escherichia coli* strains from young adults and elderly people and preliminarily examined their potencies on the occurrence of cognitive impairment and colitis in mice. Oral gavages of *Escherichia coli* strains caused cognitive impairment and colitis (Supplement Figure S4A, B). However, the difference on the occurrence of cognitive impairment and colitis among isolated *Escherichia coli* strains was not significant. Oral gavage of *Paenalcaligenes hominis* isolated from the feces of elderly people also caused cognitive impairment and colitis in mice (Supplement Figure S4C, D). However, we could not examine the difference among *Paenalcaligenes hominis* strains because it could not be isolated from young adults.

**Fig. 2:**
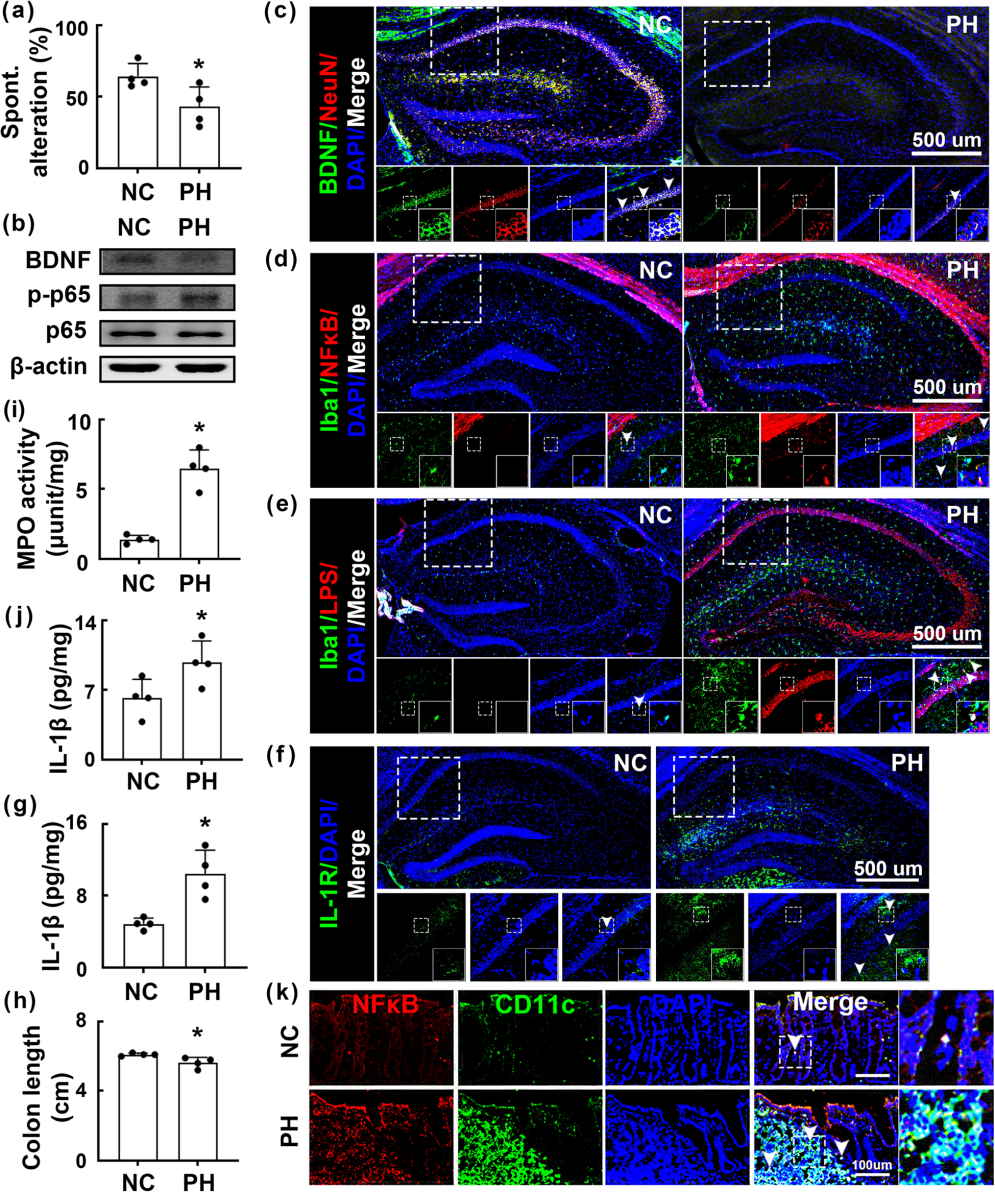
Effects of *Paenalcaligenes hominis* on the occurrence of cognitive impairment and colitis in germ-free mice. **a** Effects on the occurrence of cognitive impairment in Y-maze task. **b** Effects on the BDNF expression and NF-κB activation in the hippocampus. Effects on BDNF^+^/NeuN^+^ (**c**), NF-κB^+^/Iba1^+^ (**d**), LPS^+^/Iba1^+^ (**e**), and IL-1R^+^ cell populations (**f**) in the hippocampus. **g** Effects on the IL-1β expression in the blood, assessed by ELISA. Effects on the colon length (**h**), myeloperoxidase (MPO) activity (**i**), IL-1β level (**j**), and NF-κB^+^/CD11c^+^ cell population (**k**) in the colon. *Paenalcaligenes hominis* (PH, 1 × 10^7^ CFU/mouse/day) were orally gavaged once a day for 5 days. Control mice (NC) were treated with vehicle (saline) instead of PH. Data values were indicated as mean ± SD (*n* = 4). *p < 0.05 vs. NC group. **a**, **g**, **h**, **i** Two-tailed Mann-Whitney *U* test. **j** One-tailed Mann-Whitney *U* test

Therefore, we examined whether age-related gastrointestinal bacteria were associated with cognitive impairment and the effect of orally gavaged *Paenalcaligenes hominis* on the occurrence of cognitive impairment in germ-free mice. Exposure to this microbe caused significant cognitive impairment, including decreased spontaneous alteration in the Y-maze task and decreased BDNF expression and BNDF^+^/NeuN^+^ cell counts in the hippocampus (Fig. 2a-c, Supplement Figure S5A, B). However, exposure to the bacterium increased the NF-κB^+^/Iba1^+^, LPS^+^/Iba1^+^, and IL-1R^+^ cell populations and induced NF-κB activation (Fig. 2B,D-F, Supplement Figure S5C–E). Furthermore, exposure to *Paenalcaligenes hominis* enhanced IL-1β expression in the hippocampus (Fig. 2g). *Paenalcaligenes hominis* treatment also caused colitis, including colon shortening and an increase in the myeloperoxidase activity, IL-1β expression, and NF-κB^+^/CD11c^+^ cell population in the colon (Fig. 2h-k, Supplement Figure S5F).

**Fig. 3:**
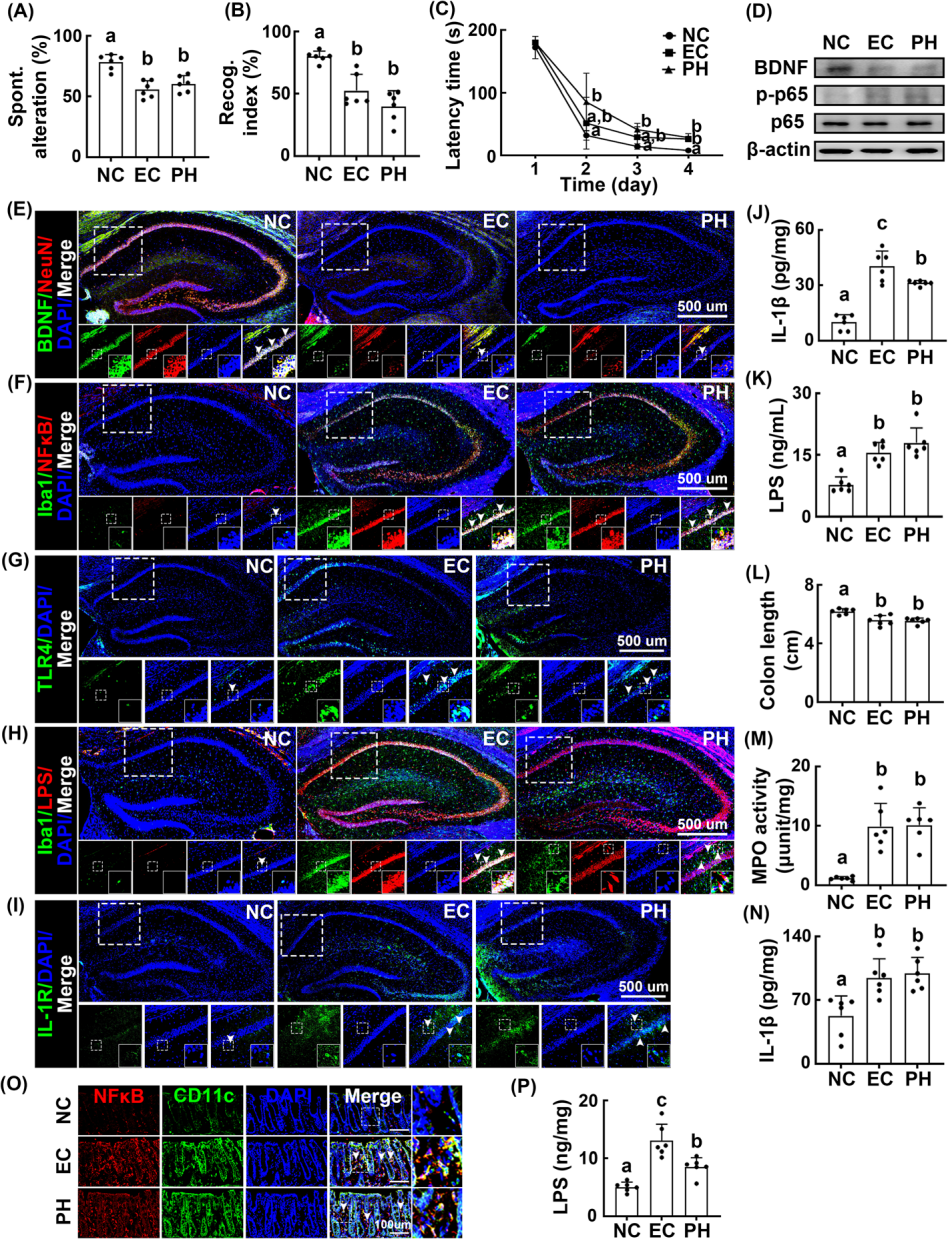
Effects of *Paenalcaligenes hominis* and *Escherichia coli* on the occurrence of cognitive impairment and colitis in specific pathogen-free mice. Effects on the occurrence of cognitive impairment in Y-maze (**A**), NOR (**B**), and Barnes maze tasks (**C**). **D** Effects on the BDNF expression and NF-κB activation in the hippocampus. Effects on the BDNF^+^/NeuN^+^ (**E**), NF-κB^+^/Iba1^+^ (**F**), TLR4^+^/Iba1^+^ (**G**), LPS^+^/Iba1^+^ (**H**), and IL-1R^+^ cell populations (**I**) into the hippocampus. **J** Effects on the IL-1β expression in the hippocampus. **K** Effects on the endotoxin levels in the blood, assessed by LAL assay kit. Effects on the colon length (**L**), myeloperoxidase (MPO) activity (**M**), IL-1β expression (**N**), and NF-κB^+^/CD11c^+^ cell population (**O**) in the colon. **P** Effects on the LPS levels in the feces. *Paenalcaligenes hominis* (PH, 1 × 10^9^ CFU/mouse/day) and *Escherichia coli* (EC, 1 × 10^9^ CFU/mouse/day) were orally gavaged for 5 days. Control mice (NC) were treated with vehicle (saline) instead of bacterial suspension. Data values were indicated as mean ± SD (*n* = 6). Means with the same letters are not significantly different (*p* < 0.05). **A**, **B**, **C**, **K**, **L**, **M**, **N** Kruskal-Wallis test with Dunn’s post hoc test for non-parametric analysis. **J**, **P** One-way ANOVA with post-hoc Bonferroni’s multiple comparisons test

Next, we orally gavaged *Paenalcaligenes hominis* or *Escherichia coli* into specific pathogen-free (SPF) mice and examined their effects on the occurrence of cognitive impairment. Oral gavage of *Paenalcaligenes hominis* or *Escherichia coli* dose-dependently impaired cognitive function in the Y-maze task (Supplement Figure S6). Treatment with *Paenalcaligenes hominis* or *Escherichia coli* at 1 × 10^9^ CFU/mouse/day significantly impaired cognitive function in the Y-maze, NOR, and Barnes maze tasks (Fig. 3A–C). These treatments also induced NF-κB activation and IL-1β expression in the hippocampus, whereas BDNF expression was suppressed (Fig. 3D, J, Supplement Figure S7A). Exposure to these bacteria also increased NF-κB^+^/Iba1^+^, toll-like receptor (TLR)4^+^/Iba1^+^, LPS^+^/Iba1^+^, and IL-1R^+^ cell populations in the hippocampus, whereas the BDNF^+^/NeuN^+^ cell population was decreased (Fig. 3E–I, Supplement Figure S7B–F). Furthermore, treatment with *Paenalcaligenes hominis* or *Escherichia coli* increased LPS levels in the blood (Fig. 3K). These bacteria additionally caused colitis, including colon shortening, myeloperoxidase activity, and IL-1β expression in the colon (Fig. 3L–N). Exposure to these bacteria increased the NF-κB^+^/CD11c^+^ cell population in the colon and fecal LPS production (Fig. 3O, P, Supplement Figure S7G).

**Fig. 4:**
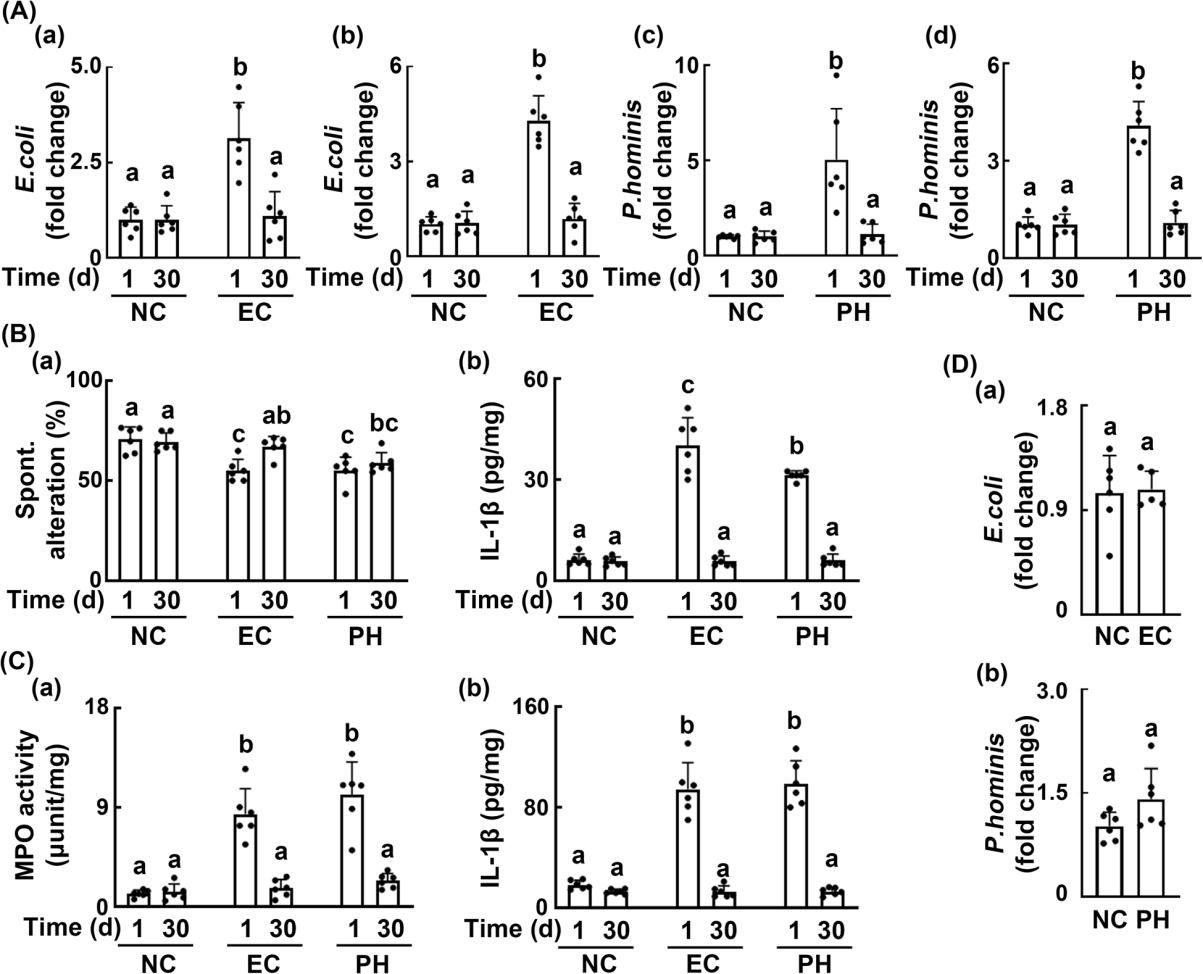
The stability of colonization with *Paenalcaligenes hominis* or *Escherichia coli* in SPF mice and their vertical transmission. **A** The colonizing stabilities of *Escherichia coli* (EC) in male (a) and female mice (b) and *Paenalcaligenes hominis* (PH) in male (c) and female mice (d). **B** The recovery of *Paenalcaligenes hominis*- or *Escherichia coli-*induced cognitive impairment (a) and IL-1β expression in the hippocampus (b) 30 days after its gavage. **C** The recovery of *Paenalcaligenes hominis*- or *Escherichia coli-*induced myeloperoxidase activity (a) and IL-1β expression in the colon (b) 30 days after its gavage. **D** Vertical transmission of *Escherichia coli* (a) or *Paenalcaligenes hominis* (b). *Paenalcaligenes hominis* or *Escherichia coli* (1 × 10^9^ CFU/mice/day) was orally gavaged for 5 days, and cognitive function was assayed in the Y-maze task 18 h after the final gavage. The feces and brain and colon tissues were collected 5 h after the final behavioral test. The offspring feces were collected on the postnatal day 14. *Paenalcaligenes hominis* or *Escherichia coli* strain-specific discrimination was performed by qPCR using DNA isolated from the fecal samples at the indicated time points (days after colonization). Control mice (NC) were treated with vehicle (saline) instead of bacterial suspension. Data values were indicated as mean ± SD (*n* = 6). Means with the same letters are not significantly different (*p* < 0.05). **A**, **B**, **C** One-way ANOVA with post hoc Bonferroni’s multiple comparisons test. **D** Two-tailed Mann-Whitney *U* test for non-parametric analysis

Next, we examined whether *Paenalcaligenes hominis* or *Escherichia coli* in SPF mice could be colonized. When *Paenalcaligenes hominis* or *Escherichia coli* was orally gavaged in mice, their populations diminished at 30 days after their final gavages to those of the control mice (Fig. 4A). The cognitive impairment of mice by oral gavage of *Paenalcaligenes hominis* or *Escherichia coli* was also recovered 30 days after the final gavage of gut bacteria to 84.7% (31.1%, compared with the cognitive function of mice at 24 h after impairment by *Paenalcaligenes hominis*) and 98.4% (92.8%, compared with the cognitive function of mice at 24 h after impairment by *Escherichia coli*) of the control mice, respectively (Fig. 4B). The recovery was slower in mice treated with *Paenalcaligenes hominis* than in those treated with *Escherichia coli*. However, the colitis was significantly recovered in mice treated with *Paenalcaligenes hominis* or *Escherichia coli*: the recovery between these groups was not significantly different (Fig. 4C). Moreover, *Paenalcaligenes hominis* and *Escherichia coli* of infected mice were not significantly transmitted to the offspring (Fig. 4D).

### Celiac vagotomy reduced the cognitive impairment induced by *Paenalcaligenes hominis*, but not by *Escherichia coli*

**Fig. 5:**
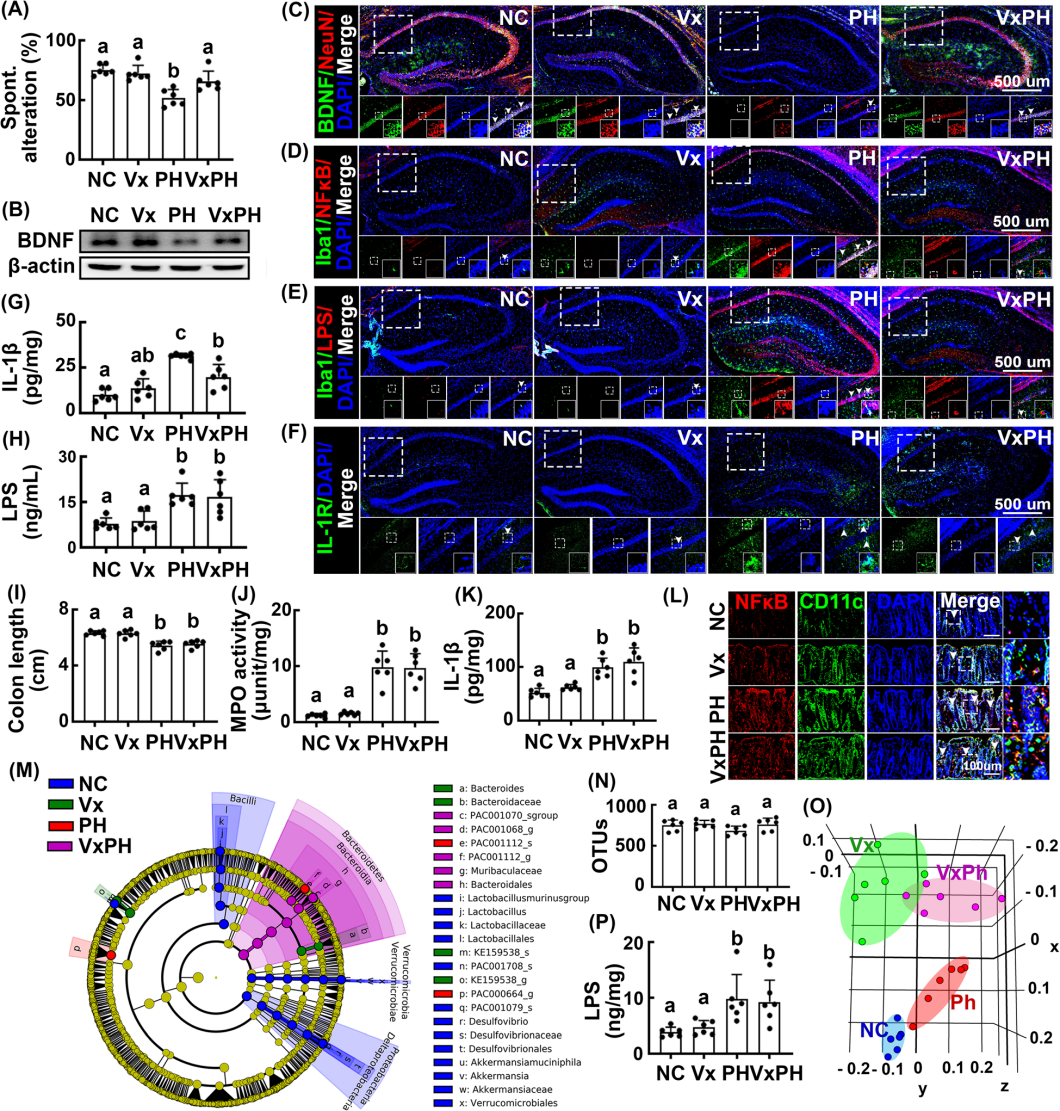
Effects of *Paenalcaligenes hominis* on the occurrence of cognitive impairment and colitis in mice with or without vagotomy. **A** Effects on the occurrence of cognitive impairment in Y-maze task. **B** Effects on the BDNF expression in the hippocampus. Effects on the BDNF^+^/NeuN^+^ (**C**), NF-κB^+^/Iba1^+^ (**D**), LPS^+^/Iba1^+^ (**E**), and IL-1R^+^ cell populations (**F**) in the hippocampus. **G** Effect on the IL-1β levels in the blood, assessed by ELISA. **H** Effect on the endotoxin levels in the blood, assessed by the LAL assay kit. Effect on the colon length (**I**), myeloperoxidase (MPO) activity (**J**), IL-1β expression (**K**), and NF-κB^+^/CD11c^+^ cell population (**L**) in the colon. Effect on the composition of gut microbiota: cladogram (**M**) generated by LEfSE indicating significant differences in gut microbial abundances among NC (blue), Vx (purple), PH (red), and VxPH (green) group; OTUs (**N**); and principal coordinate analysis (PCoA) plot based on Jensen-Shannon analysis (**O**). The threshold logarithmic score set at 4.4 and ranked. Yellow nodes represent species with no significant difference. **P** Effect on the endotoxin levels in the feces. *Paenalcaligenes hominis* (PH, 1 × 10^7^ CFU/mouse/day) were orally gavaged for 5 days in mice with or without vagotomy. Control mice with (Vx) and without vagotomy (NC) were treated with vehicle (saline) instead of bacterial suspension. Data values were indicated as mean ± SD (*n* = 6). Means with the same letters are not significantly different (*p* < 0.05). **A**, **G**, **H**, **I**, **J**, **K**, **O** One-way ANOVA with post hoc Bonferroni’s multiple comparisons test. **P** One-way ANOVA with post hoc Holm-Sidak’s multiple comparisons test

Next, to understand the mechanisms by which orally gavaged *Paenalcaligenes hominis* and *Escherichia coli* caused cognitive impairment, we examined their effects on the occurrence of cognitive impairment and colitis in mice with or without celiac vagotomy. Vagotomy weakly caused cognitive impairment, and defecation was delayed (Fig. 5A, Supplement Figure S8). However, the occurrence of cognitive impairment following oral gavage of *Paenalcaligenes hominis* was significantly inhibited by vagotomy (Fig. 5A). Vagotomy also inhibited the suppression of BDNF expression in the hippocampus by oral gavage of *Paenalcaligenes hominis* (Fig. 5B, Supplement Figure S9A). Vagotomy decreased the *Paenalcaligenes hominis*-induced NF-κB^+^/Iba1^+^, LPS^+^/Iba1^+^, and IL-1R^+^ cell populations in the hippocampus, and increased *Paenalcaligenes hominis* reduced BDNF^+^/NeuN^+^ cell populations (Fig. 5C–F, Supplement Figure S9B-E). Vagotomy suppressed *Paenalcaligenes hominis*-induced IL-1β expression in the hippocampus (Fig. 5G); however, *Paenalcaligenes hominis*-induced blood LPS levels were unaffected by vagotomy (Fig. 5H). Furthermore, vagotomy did not affect *Paenalcaligenes hominis*-induced colitis; specifically, it did not inhibit colon shortening, myeloperoxidase activity, and IL-1β expression or alter NF-κB^+^/CD11c^+^ cell populations (Fig. 5I–L, Supplement Figure S9F). Vagotomy did not affect α-diversity in the gastrointestinal microbiota compared to that in control mice as determined using pyrosequencing, whereas β-diversity was significantly shifted (Fig. 5M–O, Supplement Figure S10A, C). Vagotomy significantly altered the composition of the gastrointestinal microbiota, specifically increasing the Bacteroidetes population and decreasing the Proteobacteria and Verrucomicrobia populations. However, vagotomy did not affect the LPS production of gut bacteria. Treatment with *Paenalcaligenes hominis* also enlarged the Bacteroidetes population and reduced the Proteobacteria and Verrucomicrobia populations in mice with or without vagotomy. Furthermore, oral gavage of *Paenalcaligenes hominis* significantly increased gastrointestinal bacterial LPS production in mice with or without vagotomy (Fig. 5P).

**Fig. 6:**
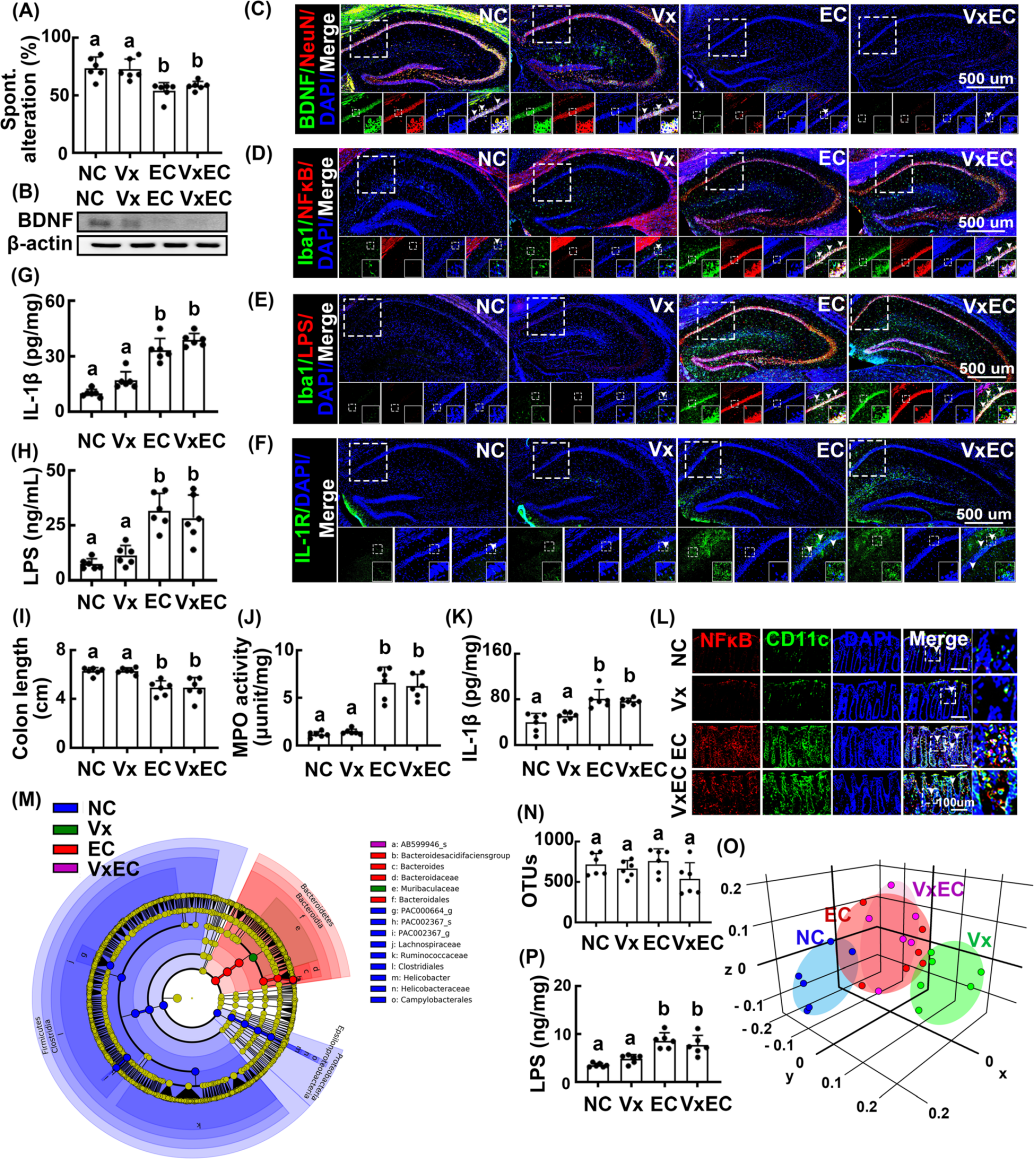
Effects of *Escherichia coli* on the occurrence of cognitive impairment and colitis in mice with or without vagotomy. **A** Effects on the occurrence of cognitive impairment in Y-maze task. **B** Effects on the BDNF expression in the hippocampus. Effects on the BDNF^+^/NeuN^+^ (**C**), NF-κB^+^/Iba1^+^ (**D**), LPS^+^/Iba1^+^ (**E**), and IL-1R^+^ cell populations (**F**) in the hippocampus. **G** Effect on the IL-1β levels in the blood, assessed by ELISA. **H** Effect on the endotoxin levels in the blood, assessed by the LAL assay kit. Effect on the colon length (**I**), myeloperoxidase (MPO) activity (**J**), IL-1β expression (**K**), and NF-κB^+^/CD11c^+^ cell population (**L**) in the colon. Effect on the composition of gut microbiota: cladogram (**M**) generated by LEfSE indicating significant differences in gut microbial abundances among NC (blue), Vx (purple), PH (red), and VxPH (green) group; OTUs (**N**); and principal coordinate analysis (PCoA) plot based on Jensen-Shannon analysis (**O**). The threshold logarithmic score set at 4.4 and ranked. Yellow nodes represent species with no significant difference. **P** Effect on the endotoxin levels in the feces. *Escherichia coli* (EC, 1 × 10^7^ CFU/mouse/day) were orally gavaged for 5 days in mice with or without vagotomy. Control mice with (Vx) and without vagotomy (NC) were treated with vehicle (saline) instead of bacterial suspension. Data values were indicated as mean ± SD (*n* = 6). Means with the same letters are not significantly different (*p* < 0.05). **A**, **B**, **G**, **H**, **I**, **J**, **K**, **N**, **P** One-way ANOVA with post hoc Bonferroni’s multiple comparisons test

Oral gavage of *Escherichia coli* caused cognitive impairment in mice without vagotomy. Vagotomy did not inhibit *Escherichia coli*-induced cognitive decline (Fig. 6A). Furthermore, vagotomy did not affect *Escherichia coli*-induced changes in NF-κB^+^/Iba1^+^, LPS^+^/Iba1^+^, BDNF^+^/NeuN^+^, and IL-1R^+^ cell populations and BDNF and IL-1β expression in the hippocampus (Fig. 6B–G, Supplement Figure S11A–E). Vagotomy also did not affect LPS levels in the blood (Fig. 6 H). Vagotomy did not affect *Escherichia coli*-induced colitis, as it did not inhibit *Escherichia coli*-induced myeloperoxidase activity, IL-1β expression, and NF-κB^+^/Iba1^+^ cell counts in the colon (Fig. 6I–L, Supplement Figure S11F). Vagotomy did not affect the α-diversity of gastrointestinal microbiota compared with the findings in control mice, whereas β-diversity was significantly shifted (Fig. 6M–O, Supplement Figure S10B, D). Vagotomy significantly altered the composition of the gastrointestinal microbiota composition, including increased abundance of Bacteroidetes and decreased abundance of Proteobacteria population. *Escherichia coli* treatment also increased the abundance of Bacteroidetes and reduced that of Proteobacteria in mice without vagotomy. *Escherichia coli* treatment also increased Bacteroidetes counts and reduced Proteobacteria counts in mice without vagotomy. Furthermore, oral gavage of *Escherichia coli* significantly increased the abundance of Bacteroidaceae and gastrointestinal bacterial LPS production in mice with or without vagotomy (Fig. 6P).

### Effects of extracellular vesicles and LPS isolated from *Paenalcaligenes hominis* in mice with or without celiac vagotomy

**Fig. 7:**
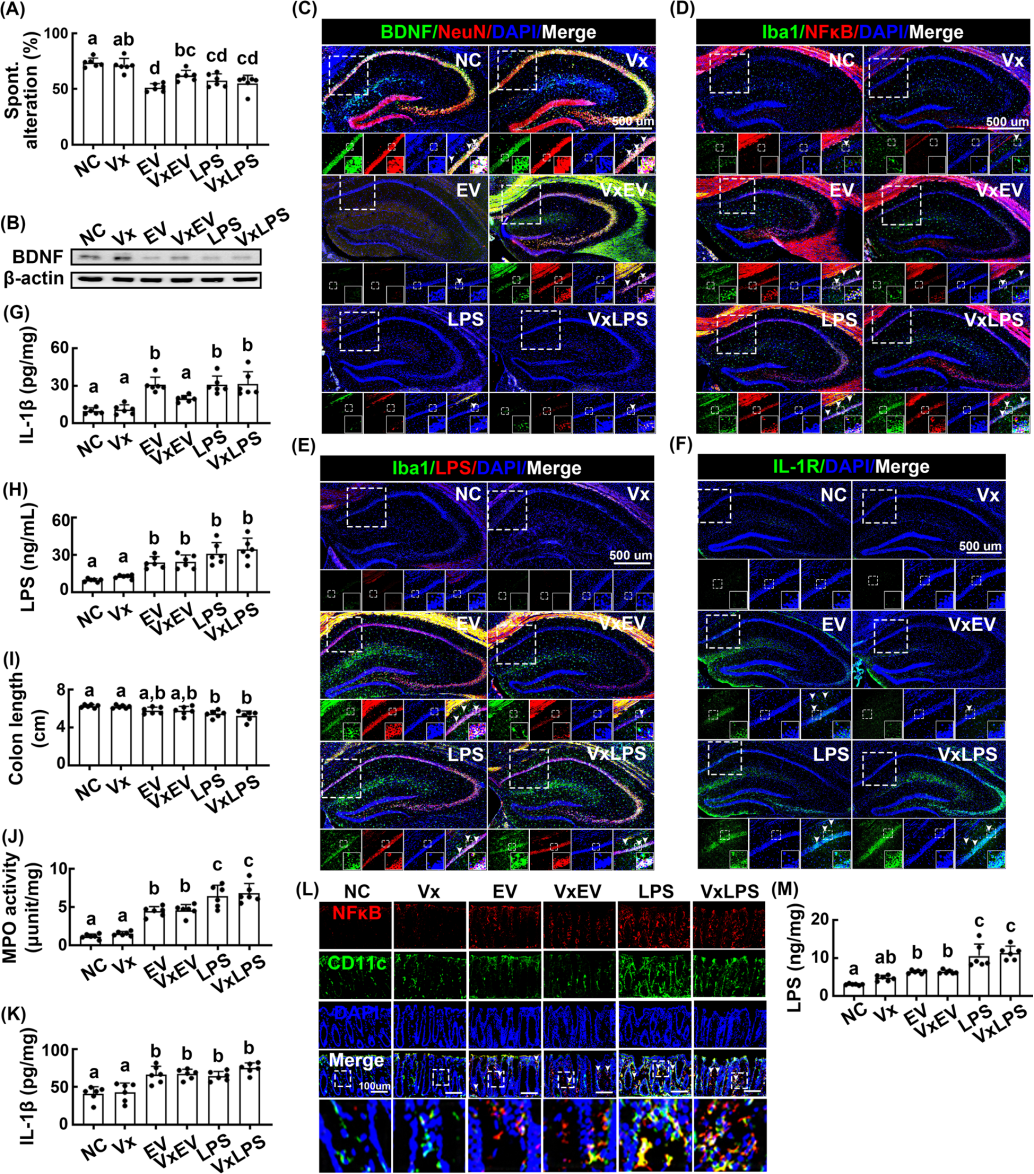
Effects of *Paenalcaligenes hominis* extracellular vesicles (EVs) and lipopolysaccharide (LPS) on the occurrence of cognitive impairment and colitis in mice with or without vagotomy. **A** Effects on the occurrence of cognitive impairment in Y-maze task. **B** Effects on the BDNF expression in the hippocampus. Effects on the BDNF^+^/NeuN^+^ (**C**), NF-κB^+^/Iba1^+^ (**D**), LPS^+^/Iba1^+^ (**E**), and IL-1R^+^ cell populations (**F**) in the hippocampus. **G** Effects on the IL-1β levels in the blood, assessed by ELISA. **H** Effects on the endotoxin levels in the blood, assessed by LAL assay kit. Effects on the colon length (**I**), myeloperoxidase (MPO) activity (**J**), IL-1β expression (**K**), and NF-κB^+^/CD11c^+^ cell population (**L**) in the colon. **M** Effects on the endotoxin levels in the feces. EV and LPS were orally gavaged for 5 days in mice with or without vagotomy. Control mice with (Vx) and without vagotomy (NC) were treated with vehicle (saline) instead of bacterial suspension. Data values were indicated as mean ± SD (*n* = 6). Means with the same letters are not significantly different (*p* < 0.05). **A**, **G**, **H**, **I**, **J**, **K**, **M** One-way ANOVA with post hoc Bonferroni’s multiple comparisons test

To identify the etiological agent responsible for celiac vagotomy reduced cognitive impairment following gavage of *Paenalcaligenes hominis*, we isolated the bacterium’s LPS fraction and examined whether it could cause cognitive impairment and whether these effects were inhibited by vagotomy. Oral gavage of LPS caused cognitive impairment in the Y-maze test, as intraperitoneally injected LPS (Fig. 7A, Supplement Figure S12A). LPS treatment also increased the numbers of NF-κB^+^/Iba1^+^, LPS^+^/Iba1^+^, and IL-1R^+^ cells in the hippocampus, whereas the BDNF expression and BDNF^+^/NeuN^+^ cell population were diminished (Fig. 7B–F, Supplement Figure S13A–E). Meanwhile, LPS treatment increased IL-1β expression in the hippocampus and LPS levels in the blood (Fig. 7G, H). However, vagotomy did not affect LPS-induced impairment of cognitive function or changes in the hippocampal NF-κB^+^/Iba1^+^, LPS^+^/Iba1^+^, and IL-1R^+^ cell counts and IL-1β expression. Vagotomy also had no effects on LPS-induced blood LPS levels. Furthermore, vagotomy did not affect LPS-induced colitis and gastrointestinal bacterial LPS production (Fig. 7I–M, Supplement Figure S13F).

Next, we isolated the EV fraction of *Paenalcaligenes hominis* and examined whether it could cause cognitive decline in mice. Oral gavage of EVs, which consisted of LPS, proteins, and nucleic acid (Supplement Methods), more potently caused cognitive impairment in mice than orally administered LPS (Fig. 7A). EV treatment also increased NF-κB^+^/Iba1^+^, LPS^+^/Iba1^+^, and IL-1R^+^ cell counts in the hippocampus (Fig. 7C–F, Supplement Figure S13C–E). EV treatment reduced BDNF expression in the hippocampus, while increasing IL-1β expression in the blood (Fig. 7B, G). EV treatment more weakly increased LPS levels in the blood and feces and the occurrence of colitis than LPS treatment (Fig. 7H, M). Vagotomy significantly reduced the occurrence of cognitive impairment caused by EV gavage (Fig. 7A). Vagotomy inhibited EV-induced changes in NF-κB^+^/Iba1^+^, LPS^+^/Iba1^+^, and IL-1R^+^ cell populations in the hippocampus and LPS levels in blood (Fig. 7C–F). However, vagotomy did not affect EV-induced colitis or blood LPS levels (Fig. 7H–L). The occurrence of cognitive impairment and colitis induced by EV treatment was weakly, but not significantly, accelerated by the addition of LPS (Supplement Figure S12A–L).

**Fig. 8:**
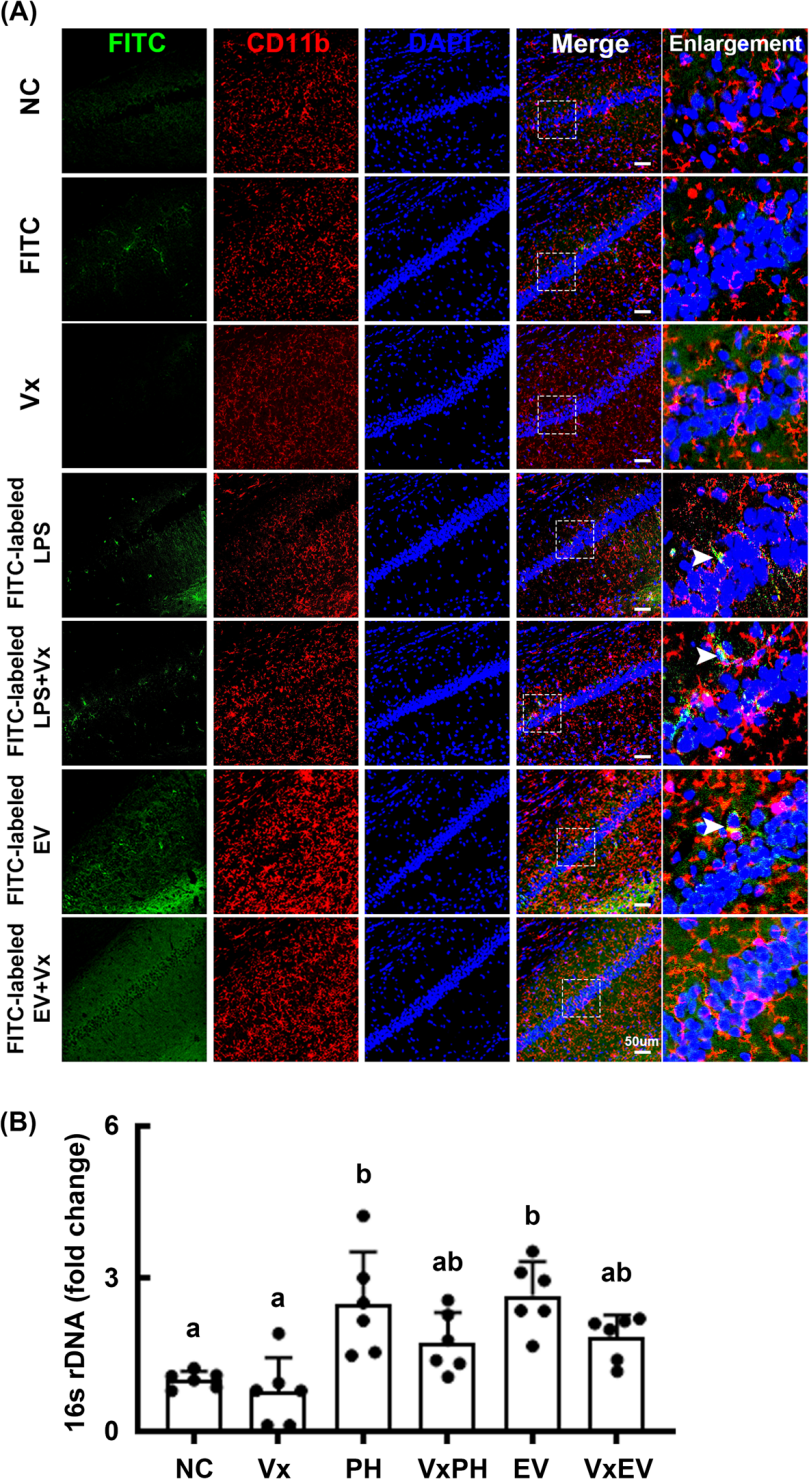
Vagotomy inhibited the accumulation of *Paenalcaligenes hominis* extracellular vesicles (EVs), but not lipopolysaccharide (LPS), in the hippocampus of mice. **A** Effect on the accumulation of FITC-labeled EV and LPS. **B** Effects on the bacterial 16S rDNA levels in the hippocampus. EV and LPS were orally gavaged for 3 days in mice with or without vagotomy. Control mice with (Vx) and without vagotomy (NC) were treated with vehicle (saline) instead of bacterial suspension. Data values were indicated as mean ± SD (*n* = 6). Means with the same letters are not significantly different (*p* < 0.05). **B** One-way ANOVA with post hoc Bonferroni’s multiple comparisons test

To confirm the mechanism by which *Paenalcaligenes hominis* LPS and EVs could induce cognitive impairment, we orally gavaged fluorescein isothiocyanate (FITC)-conjugated EVs or LPS in mice. FITC-conjugated EV and LPS, which were also detected in microglial cells, were detected in the pyramidal region of the hippocampus. FITC-conjugated EVs were more frequently detected than did FITC-conjugated LPS (Fig. 8A, Supplement Figure S14). However, vagotomy significantly reduced the FITC-conjugated EV-phagocytosed CD11c^+^ cell population, whereas it did not affect the FITC-conjugated LPS-phagocytosed CD11c^+^ cell population. We also found that oral gavage of *Paenalcaligenes hominis* or EVs increased bacterial 16S rDNA levels in the hippocampus, whereas they were inhibited by vagotomy (Fig. 8B). However, we could not observe intact EVs in the hippocampus, even by searching using transmission electron microscopy.

## Discussion

The gastrointestinal microbiota play pivotal roles in the neurodevelopmental processes and brain functions through microbiota-gut-brain (MGB) axis. Dysregulation of MGB axis by endogenous and exogenous factors, such as aging and social defeat, accelerates the occurrence of psychiatric disorders such as depression, anxiety, autism, and AD [31, 32]. Aging accelerates the incidence of neurodegenerative disorders and gut dysbiosis, which causes inflammatory bowel disease (IBD) [33, 34]. Patients with IBD display cognitive impairments and psychomotor performance, particularly during the active stage of the disease [35, 36]. The treatment of patients with IBD by an anti-inflammatory drug alleviates the brain function involved in visceral sensitivity and cognitive-affective biases [37, 38]. These findings suggest that the pathogenesis of psychiatric disorders is closely associated with the gut microbiota.

In this study, we found that the abundance of *Paenalcaligenes hominis* and *Escherichia coli* were 4.3- and 6.6-fold higher in the feces of aged mice than in the feces of young mice, respectively, and 8.7- and 7.7-fold higher in the feces of elderly individuals than in the feces of young adults, respectively, as previously reported [21, 25]. When cultured in selective medium, the number of bifidobacteria/lactobacilli cultured on glucose blood liver (BL) agar plates was smaller for feces from elderly adults and aged mice than for feces from young adults and mice, whereas the number of Enterobacteriaceae/Alcaligenaceae cultured on DHL agar plates was higher for the feces of aged mice. *Escherichia coli* and *Paenalcaligenes hominis* populations were larger in the feces of aged mice and elderly adults than in those of young mice and young adults, respectively, as previously reported [14, 23, 25, 39]. Transplantation of feces from aged mice or elderly adults, which contained *Paenalcaligenes hominis* and *Escherichia coli*, caused cognitive impairment and colitis in transplanted young mice. Kämpfer et al. detected *Paenalcaligenes hominis* in an elderly paraplegic patient with neurogenic bladder [24]. Mitsuoka reported that *Escherichia coli* counts increased with aging [14, 39]. Jang et al. reported that TNBS-inducible *Escherichia coli* caused the cognitive impairment and colitis in mice [26]. These results suggest that *Paenalcaligenes hominis* may be an opportunistic pathogen that is pestiferous in the elderly people, and *Paenalcaligenes hominis* and *Escherichia coli* might be associated with aging-dependent neuronal disorders. The potencies of *Escherichia coli* strains isolated from young adults and elderly individuals on the occurrence of cognitive impairment were not significantly different. *Paenalcaligenes hominis* and *Escherichia coli* in infected mice were not significantly transmitted to the offspring. The incidence of cognitive impairment by exposure to *Paenalcaligenes hominis* (at a dose of 1 × 10^7^ CFU/mouse/day) was more severe in germ-free mice than in SPF mice. The incidence of cognitive impairment by exposure to *Paenalcaligenes hominis* at a dose of 1 × 10^7^ CFU/mouse/day was not significantly different with that in SPF mice treated with *Paenalcaligenes hominis* at a dose of 1 × 10^9^ CFU/mouse/day. Furthermore, *Paenalcaligenes hominis* and *Escherichia coli* orally gavaged in mice were gradually expelled over time. These results suggest that gut commensal normal microbiota may protect from the infection of pathogens, including *Paenalcaligenes hominis* and *Escherichia coli*, in the gastrointestinal tract, and the induction of this bacterial growth may be influenced in the gut by intrinsic and extrinsic factors, such as aging. Moreover, the impairment of cognitive function by *Escherichia coli* significantly recovered within 1 month, whereas cognitive impairment by *Paenalcaligenes hominis* hardly recovered. These results suggest that the natural recovery of *Paenalcaligenes hominis*-induced cognitive impairment may be time-consuming or difficult. Nevertheless, further studies are required to clearly elucidate these effects.

Oral gavage of *Paenalcaligenes hominis*, *Escherichia coli*, or transplantation of feces collected from aged mice or elderly adults significantly caused colitis in mice, modified the composition of the gastrointestinal microbiota, and increased gut bacterial LPS production. Jang et al. reported that the induction of colitis by TNBS elevates the absorption of FITC-labeled dextran and fecal and blood LPS levels in mice [26]. We also found that *Paenalcaligenes hominis*, *Escherichia coli*, or aged mouse or elderly adult feces treatments significantly caused colitis and increased LPS levels in the feces and blood. These results suggest that oral gavage of the feces collected from aged mice or elderly adults, *Paenalcaligenes hominis*, or *Escherichia coli* can increase the absorption of microbial byproducts, such as LPS, into the blood due to the increase in the gastrointestinal membrane permeability induced by gastrointestinal inflammation and induction of gut bacterial LPS production.

Exposure to feces from aged mice or elderly adults, *Paenalcaligenes hominis*, or *Escherichia coli* impaired cognitive function in mice and increased the numbers of activated microglia (NF-κB^+^/Iba1^+^, TLR4^+^/Iba1^+^, and LPS^+^/Iba1^+^ cells), IL-1R^+^ cells, and IL-1β expression in the hippocampus, whereas BDNF^+^/NeuN^+^ cell counts and BDNF expression were reduced.

In particular, ratios of TLR4^+^/Iba1^+^ and LPS^+^/Iba1^+^ cell populations to BDNF^+^/NeuN^+^ cell population were higher in feces-transplanted mice than in mice treated with *Paenalcaligenes hominis*, whereas ratio of NF-κB^+^/Iba1^+^ cell population with BDNF^+^/NeuN^+^ cell population was higher in feces-transplanted mice than in mice treated with *Escherichia coli*. These results suggest that the incidence of cognitive impairment facilitated by fecal transplantation may be due to the simultaneous stimulation of many gut commensal bacteria such as *Paenalcaligenes hominis* and *Escherichia coli*. Furthermore, the occurrence of cognitive impairment induced by *Paenalcaligenes hominis* was not different between germ-free and SPF mice. Furthermore, the oral gavage of *Paenalcaligenes hominis* suppressed GABA_A_ receptor-α2 subunit expression and increased GABA_B_ receptor-1b subunit expression (Supplement Figure S15). These receptors are associated with cognitive function [40, 41]. Jang et al. also reported that the oral gavage of TNBS-inducible *Escherichia coli* increased NF-κB activation and NF-κB^+^/Iba1^+^ cell counts and reduced BDNF expression and BDNF^+^/NeuN^+^ cell counts in mice [26]. The inhibition of IL-1β expression by pretreatment with an IL-1R antagonist mitigates the neuroinflammatory effects of postoperative cognitive dysfunction in mice [42]. These results suggest that *Paenalcaligenes hominis* and *Escherichia coli* can cause hippocampal inflammation by inducing TLR4- and IL-1R-mediated NF-κB activation in innate immune cells such as microglia and successively suppress NF-κB-mediated BDNF expression in the hippocampus, resulting in cognitive impairment.

*Paenalcaligenes hominis* and *Escherichia coli* caused dose-dependent cognitive impairment in mice. Their populations were significantly higher in aged mice and elderly individuals than in young mice and adults, respectively. Mitsuoka reported that the abundance of *Escherichia coli* population was higher in elderly individuals than in pediatric and young adults [14, 39]. These results suggest that aging can increase the abundance of *Paenalcaligenes hominis* and *Escherichia coli* and the stimulation of *Paenalcaligenes hominis* and/or *Escherichia coli* growth in the gastrointestinal microbiota can suppress BDNF expression in neuronal cells by inducing IL-1R- and TLR4-mediated NF-κB activation in microglia cells. *Paenalcaligenes hominis* and *Escherichia coli* also induced gut microbiota LPS production, revealed excessive bacterial LPS in the colon, and increased NF-κB activation in the macrophages and dendritic cells, resulting in the occurrence of colitis. The induction of colitis by these bacteria increased blood LPS levels, thereby inducing systemic inflammation, including neuroinflammation. These results suggest that exposure to *Paenalcaligenes hominis* and *Escherichia coli* can cause colitis and neuroinflammation by inducing gut dysbiosis and bacterial LPS production, resulting in the incidence of cognitive impairment via MGB axis.

Celiac vagotomy did not significantly cause cognitive impairment in mice, as previously reported in rats [43]. However, vagotomy significantly inhibited the occurrence of cognitive impairment and colitis induced by oral gavage of *Paenalcaligenes hominis*, but not *Escherichia coli*. Vagotomy did not affect the occurrence of colitis induced by treatment with *Paenalcaligenes hominis* or *Escherichia coli*. Bravo et al. reported that vagotomy abolished the ameliorative effects of *Lactobacillus rhamnosus* JB-1 on stress-induced anxiety/depression in mice [44]. Bercik et al. reported that the anti-psychiatric activity of *Bifidobacterium longum* NCC3001 was inhibited by vagotomy in mice with dextran sodium sulfate-induced anxiety [45]. These results suggest that gastrointestinal bacteria and their byproducts can regulate the composition of microbiota and their byproducts in the gastrointestinal tract, which may be transported into the brain via the vagus nerve, resulting in the occurrence, development, and improvement of neuropsychiatric disorders.

Oral gavage of LPS isolated from *Paenalcaligenes hominis* caused cognitive impairment and colitis in mice, similarly as intraperitoneally injected LPS [26, 46]. However, vagotomy did not significantly inhibit these events. Oral gavage of *Paenalcaligenes hominis* EVs also caused cognitive impairment and colitis in mice. Vagotomy significantly reduced the occurrence of cognitive decline following the oral gavage of EV, whereas colitis was not inhibited. Following oral gavage, FITC-conjugated EVs more strongly accumulated in the hippocampus than FITC-conjugated LPS. The transport of FITC-conjugated LPS into the hippocampus was not blocked by vagotomy, whereas that of FITC-conjugated EVs was strongly inhibited. Furthermore, vagotomy reduced bacterial 16S rDNA levels in the hippocampus of mice orally gavaged with *Paenalcaligenes hominis* or its EVs. Oral gavage of EVs with LPS caused more severe cognitive impairment and colitis in mice. These findings suggest that the proliferation of *Paenalcaligenes hominis* in the intestine of elderly individuals and aged mice can accelerate the absorption of its LPSs into the brain via blood and translocation of its EVs via the vagus nerve, resulting in cognitive impairment induced by brain inflammation. These findings suggest that the impairment of cognitive function by *Paenalcaligenes hominis* may be attributable to the combined effects of its EVs and LPS.

## Conclusions

The transplantations of feces collected from elderly individuals and aged mice caused significantly more severe cognitive impairment with colitis in transplanted young mice than those from young adults and mice. *Paenalcaligenes hominis* and *Escherichia coli* isolated from elderly individuals and aged mice can cause colitis and disorders associated with cognitive decline, such as AD. The EVs of *Paenalcaligenes hominis* may cause cognitive decline with colitis by translocating into the brain through the blood and vagus nerve.

## Methods

### Culture of gastrointestinal bacteria

The fresh feces of elderly individuals, young adults, young mice, and aged mice (0.2 g) were collected, immediately suspended in 1.8 mL of general anaerobic medium (GAM, Nissui Pharmaceutical Inc., Tokyo, Japan) broth, inoculated onto BL and DHL agar plates (Nissui Pharmaceutical Inc.), and anaerobically cultured at 37 °C for 3 days [31]. The colonies grown in agar plates were inoculated into GAM semisolid media. To identify bacteria, Gram staining, 16S rDNA sequencing, and API kit assays were performed, as previously reported [28]. Gastrointestinal bacteria including *Paenalcaligenes hominis* and *Escherichia coli* were cultured in the GAM broth [26]. For in vitro and in vivo experiments, bacteria were anaerobically cultured in GAM broth at 37 °C (0.8–1.0 at 600 nm), centrifuged for 20 min at 5000×*g*, and washed twice with saline. Collected cells (1 × 10^10^ CFU/mL) were suspended in saline.

### Preparation of EVs and LPS from *Paenalcaligenes hominis*

The EVs of *Paenalcaligenes hominis* were isolated, as described by Kim et al. [47]. Briefly, *Paenalcaligenes hominis* was cultured in GAM broth at 37 °C for 24 h and centrifuged (5000×*g*, 4 °C, 20 min). The resulting supernatant was centrifuged (100,000×*g*, 4 °C, 2 h) using sucrose solution (0.8 and 2.5 mol/L). The collected interlayer was centrifuged at 150,000×*g* for 2 h. The resulting precipitate was used as EVs. The characteristics of EVs were analyzed via tandem mass spectrometry, sodium dodecyl sulfate-polyacrylamide gel electrophoresis, protein assays, and Limulus amebocyte lysate (LAL) assays (Supplement Figures S16 and S17, Supplement Table S1). *Paenalcaligenes hominis* LPS was purified as described previously (Supplement Methods) [26]. FITC (F7250 Sigma, Aldrich)-conjugated EVs (FITC to protein ratio, 0.01) and LPS (FITC to LPS ratio, 0.1) were prepared as described by Park et al. [48].

### Volunteers

Volunteers, consisting of young (average age, 20.0 ± 3.6 years) and elderly adults (average age, 62.0 ± 1.8 years), were recruited from Kyung Hee University (Seoul, Korea) (Supplement Table S2). Volunteers were enrolled if antibacterial medications were not received within 3 months before stool collection. The study protocol and consent forms for the collection of stool samples were approved by the Committee for the Care and Use of Clinical Study in the Medical School of Kyung Hee University (IRB no., KHUASP(SE)-18-045). All experimental procedures were conducted in compliance with the principles of the Declaration of Helsinki and Korean Good Clinical Practice guidelines.

### Animals

SPF C57BL/6 mice (male, 5 weeks old, 18–22 g; male, 8 weeks old, 21-24 g; female, 8 weeks old, 21–24 g) were obtained from Koatech Inc. (Seoul, Korea). SPF C57BL/6 mice (male, 6 weeks and 18 months old) were purchased from Raonbio Inc. (Yongin, Gyunggi-do, Korea). Mice were kept in wire cages under a ventilated condition (3 mice/cage, 20–22 °C, 50% ± 10% humidity, and 12-h/12-h light/dark cycle) and fed standard laboratory chow and water ad libitum. Germ-free C57BL/6J mice (male, 18–21 g, 5 weeks old) were purchased from Clea Japan Inc. (Tokyo, Japan). The mouse breeding protocol is described in the Supplementary information. Germ-free mice were housed in flexible film plastic isolators. All conditions were kept sterile in accordance with The Guidelines for Laboratory Germ-free Animals Care and Usage. Mice were used in the experiments after acclimation for 1 week. All animal experiments were approved by the Institutional Animal Care and Use Committee of Kyung Hee University (IACUC No., KUASP(SE)-17-128, 18-115, and 19-290) and performed according to the NIH and University Guide for Laboratory Animals Care and Usage.

### Treatment with gastrointestinal bacteria, EVs, and LPS in mice

*Paenalcaligenes hominis*, *Escherichia coli* (1 × 10^7^, 1 × 10^8^, or 1 × 10^9^ CFU/mouse/day), fecal suspension (25 mg/kg/day, suspended in saline), bacterial EVs with or without FITC (EVs containing 10 μg/kg protein and 32 ng/kg LPS per day, suspended in 0.2 mL of saline), and LPS with or without FITC (100 μg/kg/day as LPS, suspended in saline) were orally gavaged daily for 5 days in mice with or without celiac vagotomy (Supplement Figure S18). LPS (8 μg/kg/day, LPSip) was also intraperitoneally injected in mice once a day for 5 days. Each group consisted of six mice (male, 6 weeks old). The fecal suspension was prepared as follows: fresh feces from aged mice (6 weeks and 20 months old) were collected, suspended in GAM broth on ice, centrifuged at 3000×*g* for 15 min at 4 °C, washed with saline, and suspended in saline.

Memory-related behavioral tasks were performed on the fifth day after treatment with fecal or bacterial suspension in the Y-maze, NOR, and Barnes maze tests. Mice were anesthetized with alfaxalone (100 mg/kg, intraperitoneal injection: Careside, Gyeonggi-do, Korea). Colons and brains were removed in mice transcardially perfused with or without 4% paraformaldehyde.

### Celiac vagotomy

Celiac vagotomy was performed as previously reported [49]. To prepare mice for celiac vagotomy, we incised the central abdomen of each mouse to expose the front wall of the esophagus and subdiaphragmatically transected the celiac branch of the vagus nerve. To prepare a sham group, mice underwent incision of the central abdomen without vagotomy.

### Behavioral tasks

The Y-maze task was performed in a three-arm horizontal maze (40 cm long and 3 cm wide with 12-cm-high walls) according to the method of Kim et al. [50]. A mouse was initially placed within one arm, and the sequence and the number of arm entries were manually recorded for 8 min. The spontaneous alternation was defined as entries into all three arms on consecutive choices and was calculated as the ratio (%) of the actual to possible alternations. The NOR task was performed in the open field box (45 × 45 × 45 cm) made using black acrylic panel according to the method of Kim et al. [50]. In the first trial, a mouse was placed in the box containing two identical objects and the frequency of touching each object was recorded for 10 min. The second trial was conducted 24 h after the first trial; a mouse was placed in the box containing one of the old objects, which was used in the first trial, and a new object. NOR was calculated as the ratio of the frequency of touching the new object to the sum of the touching frequencies. The Barnes maze task was performed in the maze consisted of a circular platform (diameter, 89 cm) with 20 holes (diameter, 5 cm) situated evenly around the perimeter and an escape box, which was located below the platform, according to the method of Kim et al. [50]. The training/acquisition phase finished after the mouse entered the escape box or after the maximum test duration (5 min), following which the mouse was allowed to stay in the box for 30 s. If the mouse failed to enter the escape box within 5 min, it was led to the escape box. Mice were given two trials each day for 5 consecutive days.

### Immunofluorescence assay

The brains and colons of mice were transcardially perfused with 4% paraformaldehyde, post-fixed with 4% paraformaldehyde for 4 h, cytoprotected in 30% sucrose solution, frozen, and sectioned. Immunostaining for the sectioned tissues was performed as previously reported [28]. Briefly, tissue sections were washed with phosphate-buffered saline, blocked with normal serum, and incubated with antibodies against NeuN (1:200, Millipore: cat #MAB377), BDNF (1:50, Santa Cruz Biotechnology: cat # SC-65513), NF-κB (p-p65, 1:100, Cell Signaling Technology: cat # 3033S), LPS (1:100, Abcam: cat #ab35654), Iba1 (1:200, Thermo Fisher Scientific: cat #PA5-27436), TLR4 (1:50, Santa Cruz Biotechnology: cat #SC-293072), IL-1R (1:100, Abcam: cat #ab106278), and/or CD11c (1:100, Abcam: cat #ab11029) overnight, followed by incubation with secondary antibodies conjugated with Alexa Fluor 594 (1:200, Invitrogen) or Alexa Fluor 488 (1:200, Invitrogen) for 2 h. Nuclei were stained with 4′,6-diamidino-2-phenylindole, dilactate (Sigma Aldrich: cat #F6057). Immunostained samples were observed using a confocal laser microscope.

### Enzyme-linked immunosorbent assay and immunoblotting

Brain and colon tissues were homogenized with radioimmunoprecipitation assay lysis buffer (Biosesang Inc., Seongnam, Korea: cat #RC2002) containing a phosphatase inhibitor cocktail and 1% protease inhibitor cocktail on ice [28]. For the enzyme-linked immunosorbent assay (ELISA) assay, the supernatants were transferred into 96-well plates, and the animals’ cytokine levels were determined using ELISA kits (eBioscience, San Diego, CA, USA) [28]. For the immunoblotting assay, tissue lysate supernatants were electrophoresed using sodium dodecyl sulfate-polyacrylamide gel electrophoresis and transferred to a nylon membrane. Proteins were visualized using primary and secondary antibodies [25].

### Myeloperoxidase activity and LAL assays

Myeloperoxidase activity was assayed as previously reported [28]. Fecal and blood endotoxin levels were assayed using an LAL assay kit (Cape Cod Inc., E. Falmouth, MA: cat # C1500) [51].

### Pyrosequencing

The fresh stools of five mice were collected, and their bacterial genomic DNAs were extracted using a commercial DNA isolation kit (QIAamp DNA stool mini kit), as previously reported [50, 52]. Amplification of genomic DNA was performed using barcoded primers targeted the bacterial 16S rRNA V4 region gene. Each amplicon was sequenced using Illumina iSeq 100 (San Diego, CA). Prediction for functional genes was analyzed using the phylogenetic investigation of communities by reconstruction of unobserved states (PICRUSt) [53]. Linear discriminant analysis (LDA) and cladograms were pictured using the LDA effect size (LefSe) on Galaxy platform (https://huttenhower.sph.harvard.edu/galaxy/) [54]. The pyrosequencing reads were deposited in the short read archive of NCBI under accession number PRJNA598789.

### Quantitative real-time polymerase chain reaction

Quantitative real-time polymerase chain reaction (qPCR) for *Paenalcaligenes hominis*, *Escherichia coli*, and 16S rRNA was performed on the Rotor-Gene Q® using DNA polymerase and SYBR Green I (Takara Bio Inc.: RR820A) as previously reported [28]. Thermal cycling was performed at 95 °C for 30 s followed by 42 cycles of denaturation at 95 °C for 5 s, annealing at 55ºC for 30 s and extension at 72 °C for 30 s. Gene expression was calculated relative to 16S rDNA expression using Microsoft Excel. Primers for qPCR are indicated in Supplement Tables S3 and S4. Normalization of expression for each target gene to that of glyceraldehyde 3-phosphate dehydrogenase was computed for all samples using Microsoft Excel.

### Statistics

Experimental data are described as the mean ± SD using GraphPad Prism 8 (GraphPad Software, Inc., San Diego, CA, USA). Significant differences were analyzed using one-way ANOVA with post hoc Bonferroni’s or Holm-Sidak’s multiple comparisons test, one- or two-tailed Mann-Whitney *U* test for non-parametric test, and non-parametric  ANOVA with Kruskal-Wallis test and  Dunn’s post hoc test for non-parametric analysis (*p* < 0.05). All data related to the accumulated effects of *Paenalcaligenes hominis* and *Escherichia coli* on the occurrence of cognitive impairment in the Y-maze task were indicated in Supplement Figure S19. All *p* values are indicated in Supplement Table S5.

## Supplementary information

**Additional file 1: Figure S1**. The number of fecal bacterial colonies grown in Bifidobacteria/lactobacilli-selective BL and Enterobacteriaceae-selective DHL agar plates and *Paenalcageligenes hominis* and *Escherichia coli* populations in the feces. **Figure S2**. Effects of young and aged mouse fecal transplantations on the occurrence of cognitive impairment and colitis in the transplanted mice. **Figure S3**. Intensities of Fig. 1 immunoblotting and confocal microscope data. **Figure S4.** Effects of Escherichia coli strains isolated from the feces of young adult, elderly individual, and young mice, and aged mice and a Paenalcaligenes hominis strain isolated from the feces of elderly individual on the occurrence of cognitive impairment and colitis in mice. **Figure S5**. Intensities of Fig. 2 immunoblotting and confocal microscope data. **Figure S6**. *Paenalcaligenes hominis* (A) and *Escherichia coli* (B) dose-dependently caused cognitive impairment in specific pathogen-free mice in Y-maze task. **Figure S7**. Intensities of Fig. 3 immunoblotting and confocal microscope data. **Figure S8**. Vagotomy delayed the defecation in mice with (Vx) or without vagotomy (NC). **Figure S9**. Intensities of Fig. 5 immunoblotting and confocal microscope data. **Figure S10.** Effects of *Paenalcaligenes hominis* and *Escherichia coli* on the gut microbiota composition in mice with or without vagotomy. **Figure S11**. Intensities of Fig. 6 immunoblotting and confocal microscope data. **Figure S12.** Effects of *Paenalcaligenes hominis* extracellular vesicles (EVs) and/or lipopolysaccharide (LPS) on the occurrence of cognitive impairment and colitis in mice with or without vagotomy. **Figure S13**. Intensities of Figure 7 immunoblotting and confocal microscope data. **Figure S14**. Intensities of Figure 8A confocal microscope data. **Figure S15**. *Paenalcaligenes hominis* and *Escherichia coli* on the expression of GABA and NMDA receptors in the hippocampus. **Figure S16.** Transmission electron microscope image of *Paennalcaligenes hominis* (PH) extracellular vesicle (EV). **Figure S17**. Sodium-polyacrylamide gel electrophoresis of intact PH and EV (A, B, and C, shown in Table S1). **Figure S18**. Protocols of in vivo experiments. **Figure S19**. Accumulated effects of *Paenalcaligenes hominis* with or without vagotomy and *Escherichia coli* with or without vagotomy on the occurrence of cognitive impairment in the Y-maze task. **Table S1**. LC-MS-MS data of EV A, B, and C proteins. **Table S2.** Clinical characteristics of study participants. **Table S3**. Primers for the qPCR of *Escherichia coli* and *Paenalcaligenes hominis.***Table S4**. Primers for qPCR. **Table S5**. P values of experimental data. **Methods 1.** Mouse breeding and gut bacteria *Paenalcaligenes hominis* and *Escherichia coli* assay. 2. Purification of LPS from *Paenalcaligenes hominis* (PH). 3. Properties of extracellular vesicles purified from *Paennalcaligenes hominis* (PH). 4. Quantitative real time – polymerase chain reaction (qPCR) for GABA receptors

## Data Availability

All the necessary data except pyrosequencing reads are included in the article. Pyrosequencing reads were deposited in the NCBI’s short read archive under accession number PRJNA598789. Further data will be shared by request.

## Abbreviations

AD: Alzheimer’s disease
BDNF: Brain-derived neurotrophic factor
DHL: Deoxycholate hydrogen sulfide lactose
ELISA: Enzyme-linked immunosorbent assay
EV: Extracellular vesicle
FITC: Fluorescein isothiocyanate
GAM: General anaerobic medium
HPA: Hypothalamic-pituitary-adrenal
LAL: Limulus amoebocyte lysate
LPS: Lipopolysaccharide
MGB: Microbiota-gastrointestinal-brain
NF: Nuclear factor
NOR: Novel object recognition
qPCR: Quantitative real-time polymerase chain reaction
TLR: Toll-like receptor
TNBS: 2,4,6-Trinitrobenzenesulfonic acid
TNF: Tumor necrosis factor
