# Supplementary material for: The extracellular vesicle of gut microbial Paenalcaligenes hominis is a risk factor for vagus nerve-mediated cognitive impairment
Source: Microbiome. 2020 Jul 15;8:107. doi: 10.1186/s40168-020-00881-2 (PMC7364628; doi:10.1186/s40168-020-00881-2)
Supplement: Supplementary file 2 — Additional file 1: Figure S1. The number of fecal bacterial colonies grown in Bifidobacteria/lactobacilli-selective BL and Enterobacteriaceae-selective DHL agar plates and Paenalcageligenes hominis and Escherichia coli populations in the feces. Figure S2. Effects of young and aged mouse fecal transplantations on the occurrence of cognitive impairment and colitis in the transplanted mice. Figure S3. Intensities of Fig. 1 immunoblotting and confocal microscope data. Figure S4. Effects of Escherichia coli strains isolated from the feces of young adult, elderly individual, and young mice, and aged mice and a Paenalcaligenes hominis strain isolated from the feces of elderly individual on the occurrence of cognitive impairment and colitis in mice. Figure S5. Intensities of Fig. 2 immunoblotting and confocal microscope data. Figure S6. Paenalcaligenes hominis (A) and Escherichia coli (B) dose-dependently caused cognitive impairment in specific pathogen-free mice in Y-maze task. Figure S7. Intensities of Fig. 3 immunoblotting and confocal microscope data. Figure S8. Vagotomy delayed the defecation in mice with (Vx) or without vagotomy (NC). Figure S9. Intensities of Fig. 5 immunoblotting and confocal microscope data. Figure S10. Effects of Paenalcaligenes hominis and Escherichia coli on the gut microbiota composition in mice with or without vagotomy. Figure S11. Intensities of Fig. 6 immunoblotting and confocal microscope data. Figure S12. Effects of Paenalcaligenes hominis extracellular vesicles (EVs) and/or lipopolysaccharide (LPS) on the occurrence of cognitive impairment and colitis in mice with or without vagotomy. Figure S13. Intensities of Figure 7 immunoblotting and confocal microscope data. Figure S14. Intensities of Figure 8A confocal microscope data. Figure S15. Paenalcaligenes hominis and Escherichia coli on the expression of GABA and NMDA receptors in the hippocampus. Figure S16. Transmission electron microscope image of Paennalcaligenes hominis (PH) extracellu [file 40168_2020_881_MOESM1_ESM.docx]

**[Supplementary information]**

**The extracellular vesicle of gut microbial *Paenalcaligenes hominis* is a risk factor for vagus-nerve-mediated cognitive impairment**


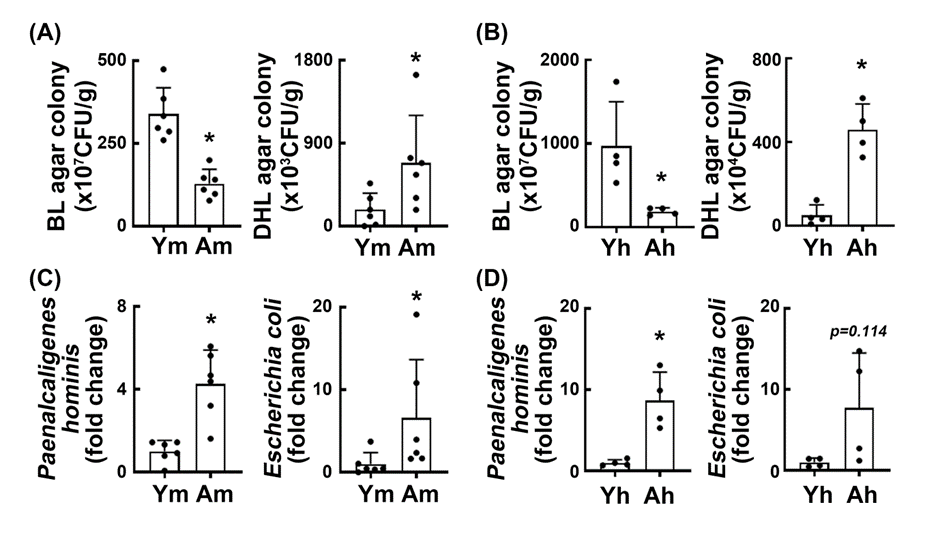


**Figure S1**. The number of fecal bacterial colonies grown in Bifidobacteria/lactobacilli-selective BL and Enterobacteriaceae-selective DHL agar plates and *Paenalcageligenes hominis* and *Escherichia coli* populations in the feces. The feces of young (Ym) and aged mice (Am), young adults (Yh), and elderly (Ah) (0.2 g) were collected, carefully suspended in the 9-volumes of dilution GAM broth on ice, diluted 10-fold in a stepwise manner, and inoculated directly in agar plates of blood liver medium (BL, Bifidobacteria-selective medium, Nissui Pharm, Japan) and hydrogen sulfate lactose medium (DHL, Enterobacteriaceae-selective medium, Eiken Chem, Japan). DHL agar plates were cultured aerobically for 1 day at 37°C and BL agar plates were cultured anaerobically for 3 days at 37°C. *Paenalcageligenes hominis* and *Escherichia coli* populations were analyzed by quantitative PCR. Data values were indicated as mean ± SD. *p < 0.05 vs. Am or Yh group. A, C, and D, two tailed Mann-Whitney U test; B, one tailed Mann-Whitney U test.


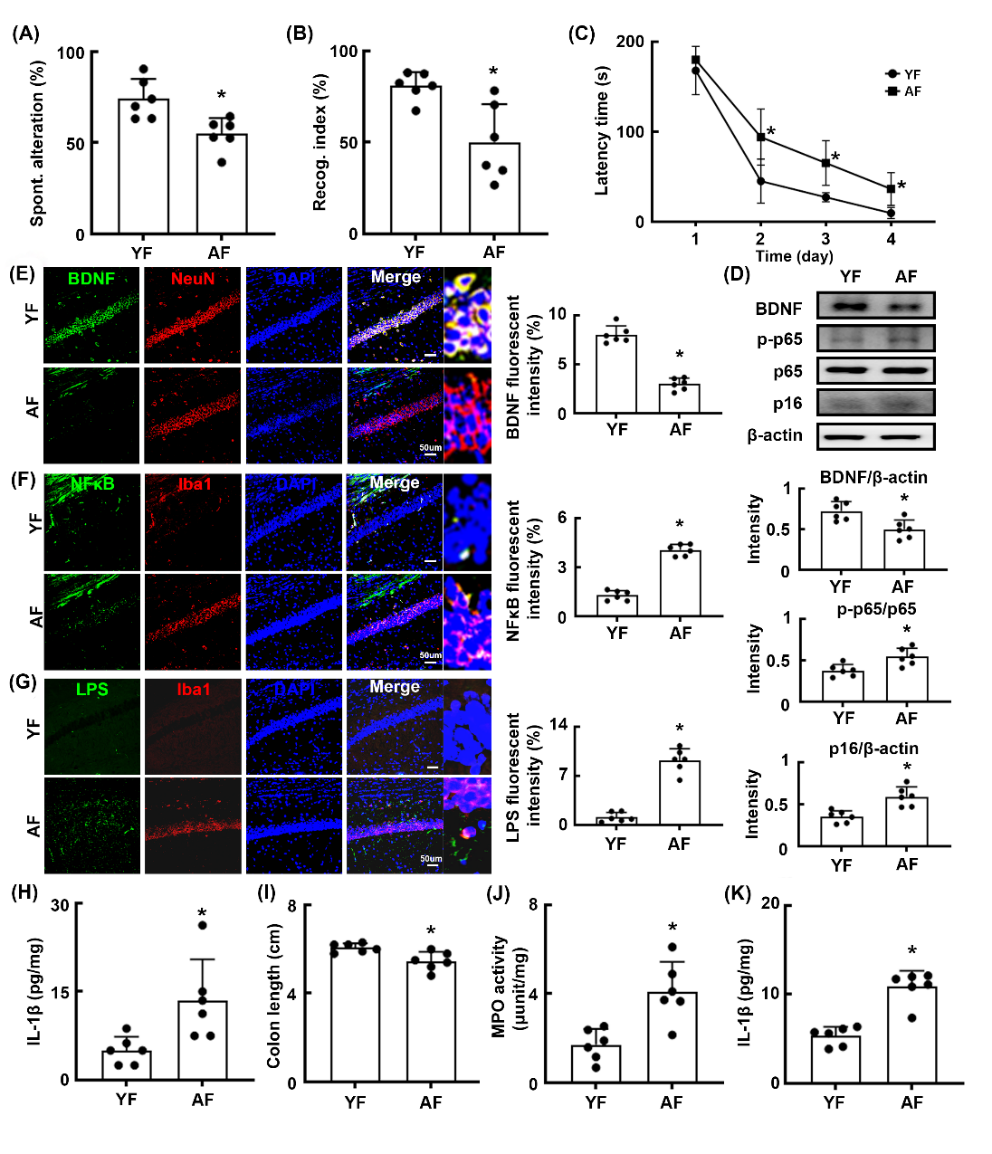


**Figure S2**. Effects of young and aged mouse fecal transplantations on the occurrence of cognitive impairment and colitis in the transplanted mice. Effects on the occurrence of cognitive impairment in Y-maze (A), novel object recognition (B), and Barnes maze tasks (C). (D) Effects on the BDNF expression and NF-κB activation in the hippocampus. Effects on the infiltration of BDNF^+^/NeuN^+^ (E), NF-κB^+^/Iba1^+^ (F), and LPS^+^/Iba1^+^ cells (G) into the hippocampus. (H) Effects on the IL-1β expression in the hippocampus, assessed by ELISA. Effects on the colon length (I), myeloperoxidase (MPO) activity (J), and IL-1β expression (K) in the colon. Fecal transplantations of young (YF) and aged mice (AF) were performed daily for 5 days. Data values were indicated as mean ± SD (n = 6). *p < 0.05 vs. YF group. A, B, C, intensities of D, E, F, and G, H, I, J , and K, two tailed Mann-Whitney U test.


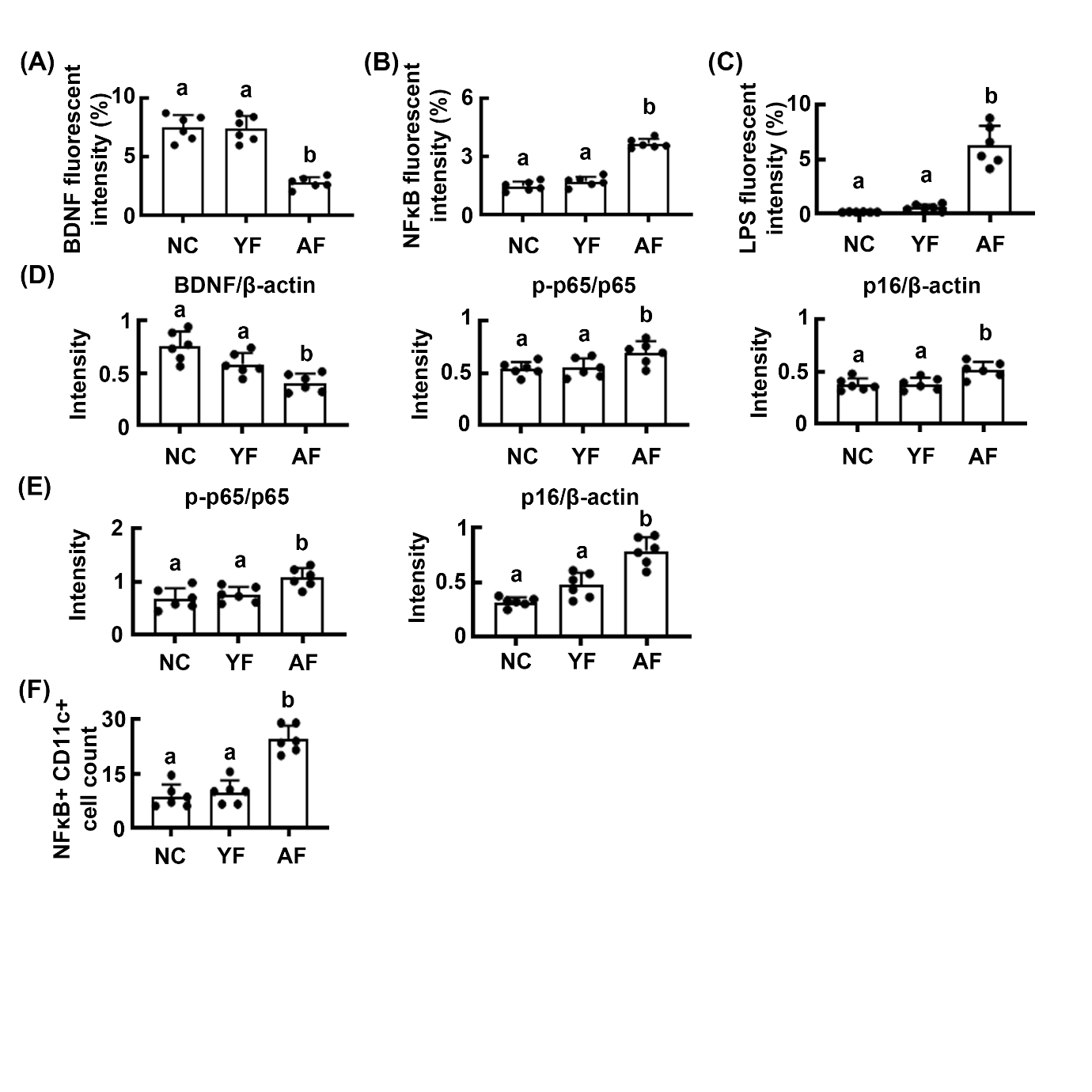


**Figure S3**. Intensities of Figure 1 immunoblotting and confocal microscope data. (A) Intensity of Figure 1D. (B) Intensity of Figure 1E. (C) Intensity of Figure 1F. (D) Intensity of Figure 1G. (E) Intensity of Figure 1L. (F) Intensity of Figure 1M. Data values were indicated as mean ± SD (n = 6). Means with same letters are not significantly different (p < 0.05). A-F, one-way ANOVA with post-hoc Bonferroni's multiple comparisons test.


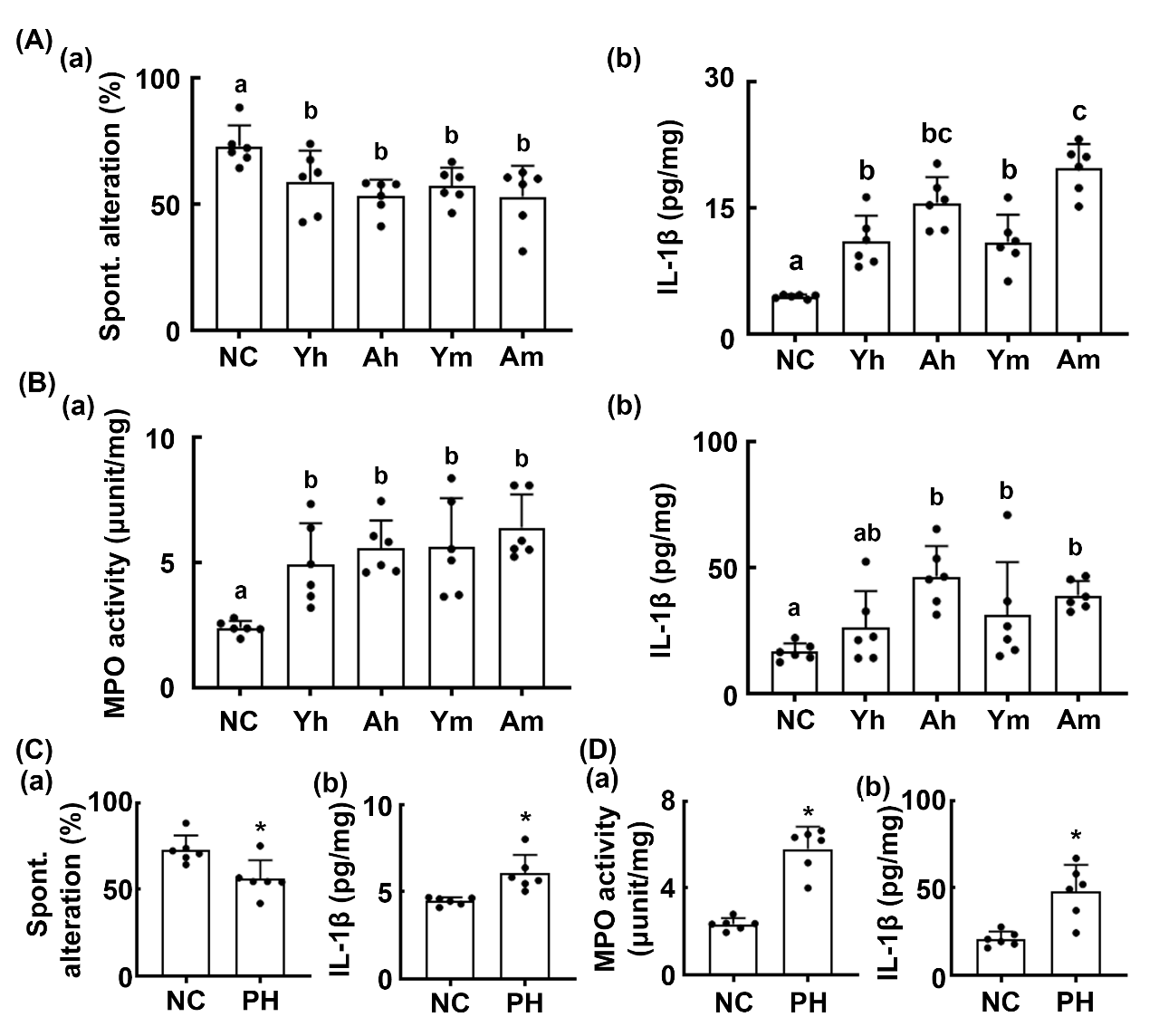
Figure S4. Effects of *Escherichia coli* strains isolated from the feces of young adult (Yh), elderly individual (Ah), and young mice (Ym), and aged mice (Am) and a *Paenalcaligenes hominis* strain isolated from the feces of elderly individual on the occurrence of cognitive impairment and colitis in mice. (A) Effect of *Escherichia coli* strains on the cognitive behaviors (a) and hippocampal IL-1β expression. (B) Effects of *Escherichia coli* strains on the myeloperoxidase activity (a) and IL-1β expression in the colon (b). (C) Effect of a *Paenalcaligenes hominis* strain isolated from the feces of elderly individual on the on the cognitive behaviors (a) and hippocampal IL-1β expression. (D) Effects of a *Paenalcaligenes hominis* strain on the on the myeloperoxidase activity (a) and IL-1β expression in the colon (b). Cognitive function was measured in the Y-maze test. *Escherichia coli* (1×10^9^ CFU/mouse/day) and *Paenalcaligenes hominis* (1×10^9^ CFU/mouse/day) were orally gavaged daily for 5 days. Control mice (NC) were treated with vehicle (saline) instead of bacterial suspension. Data values were indicated as mean ± SD (n = 6). Means with same letters are not significantly different (p < 0.05). A and B, one-way ANOVA with post-hoc Bonferroni's multiple comparisons test; C and D, two tailed Mann-Whitney U test for non-parametric analysis.


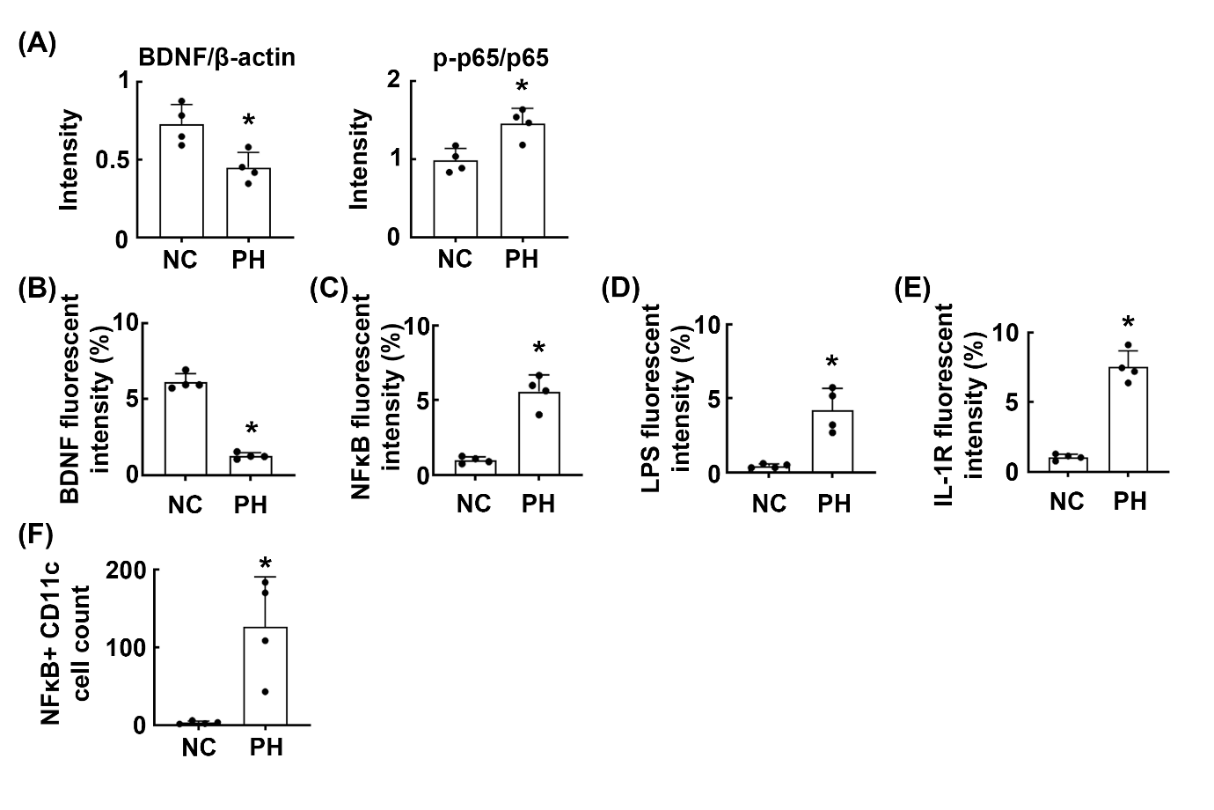


**Figure S5**. Intensities of Figure 2 immunoblotting and confocal microscope data. (A) Intensity of Figure 2B. (B) Intensity of Figure 2C. (C) Intensity of Figure 2D. (D) Intensity of Figure 2E. (E) Intensity of Figure 2F. (F) Intensity of Figure 2K. Data values were indicated as mean ± SD (n = 6). Means with same letters are not significantly different (p < 0.05). A-F, two tailed Mann-Whitney U test.


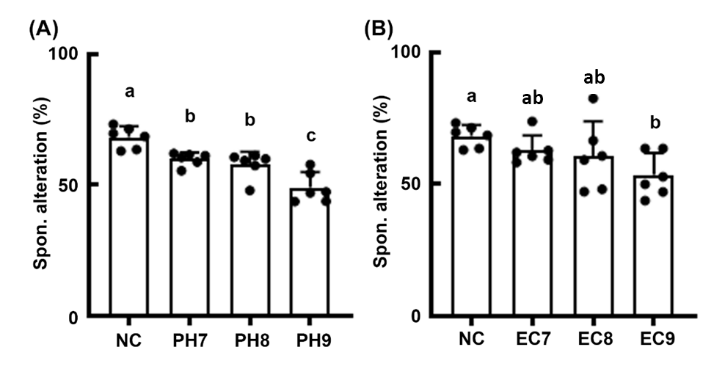


**Figure S6**. *Paenalcaligenes hominis* (A) and *Escherichia coli* (B) dose-dependently caused cognitive impairment in specific pathogen-free mice in Y-maze task. *Escherichia coli* (EC7, 1×10^7^; EC8, 1×10^8^; and EC9, 1×10^9^ CFU/mouse/day) and *Paenalcaligenes hominis* (PH7, 1×10^7^; PH8, 1×10^8^; and PH9, 1×10^9^ CFU/mouse/day) were orally gavaged daily for 5 days. Control mice (NC) were treated with vehicle (saline) instead of bacterial suspension. Data values were indicated as mean ± SD (n = 6). Means with same letters are not significantly different (p < 0.05). A and B, one-way ANOVA with post-hoc Holm-Sidak's multiple comparisons test.


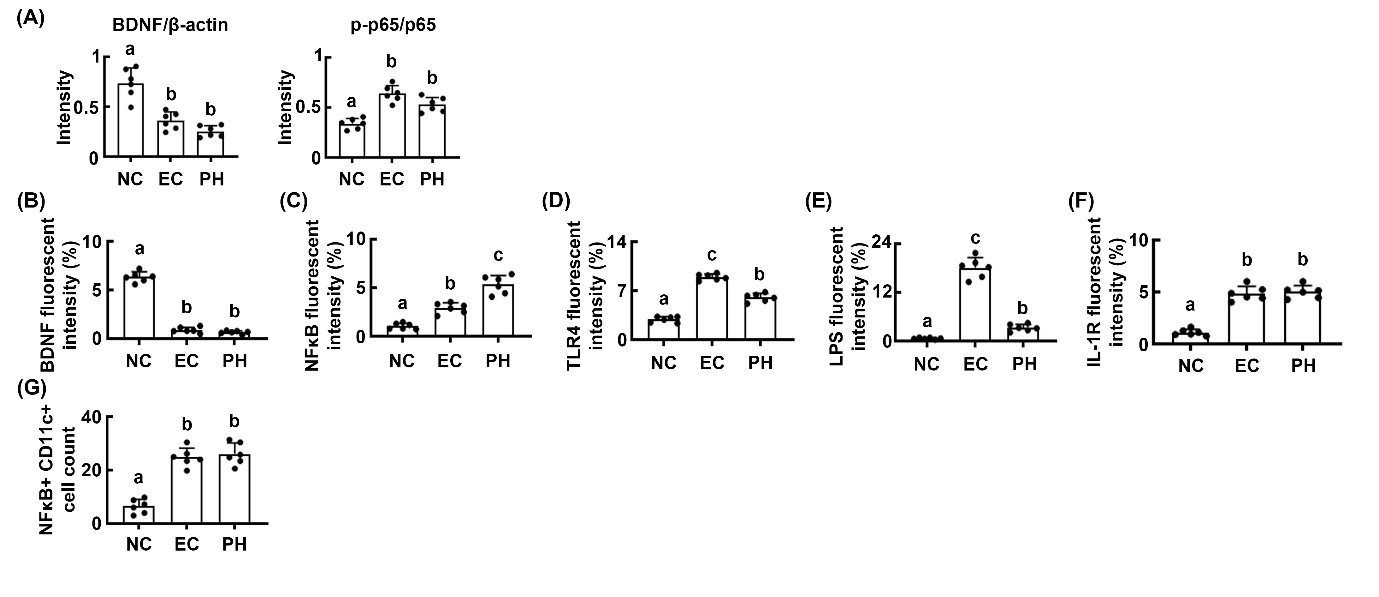


**Figure S7**. Intensities of Figure 3 immunoblotting and confocal microscope data. (A) Intensity of Figure 3D. (B) Intensity of Figure 3E. (C) Intensity of Figure 3F. (D) Intensity of Figure 3G. (E) Intensity of Figure 3H. (F) Intensity of Figure 3I. (G) Intensity of Figure 3O. Data values were indicated as mean ± SD (n = 6). Means with same letters are not significantly different (p < 0.05). A-G, one-way ANOVA with post-hoc Bonferroni's multiple comparisons test.

**
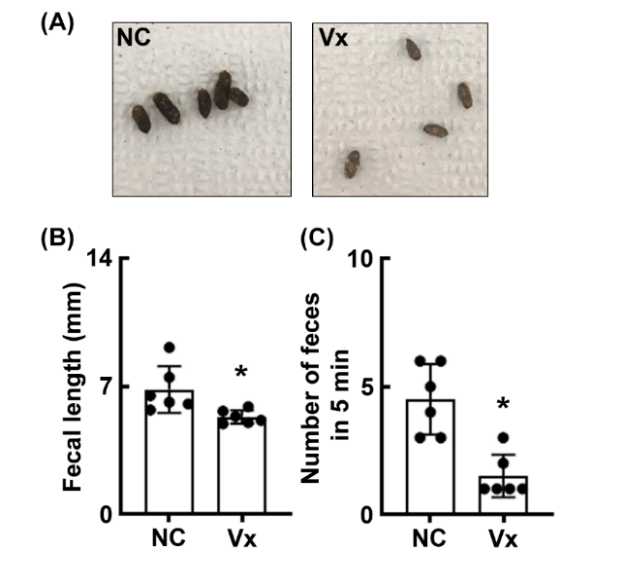
**

**Figure S8**. Vagotomy delayed the defecation in mice with (Vx) or without vagotomy (NC). (A) Photos of mouse feces. (B) Fecal length (mm). (C) Number of feces in 5 min. Vx, vagotomy-operated mice; NO, control mice. Data values were indicated as mean ± SD (n = 6). *p < 0.05 vs. NC group. C and D, one-way ANOVA with post-hoc Bonferroni's multiple comparisons test.


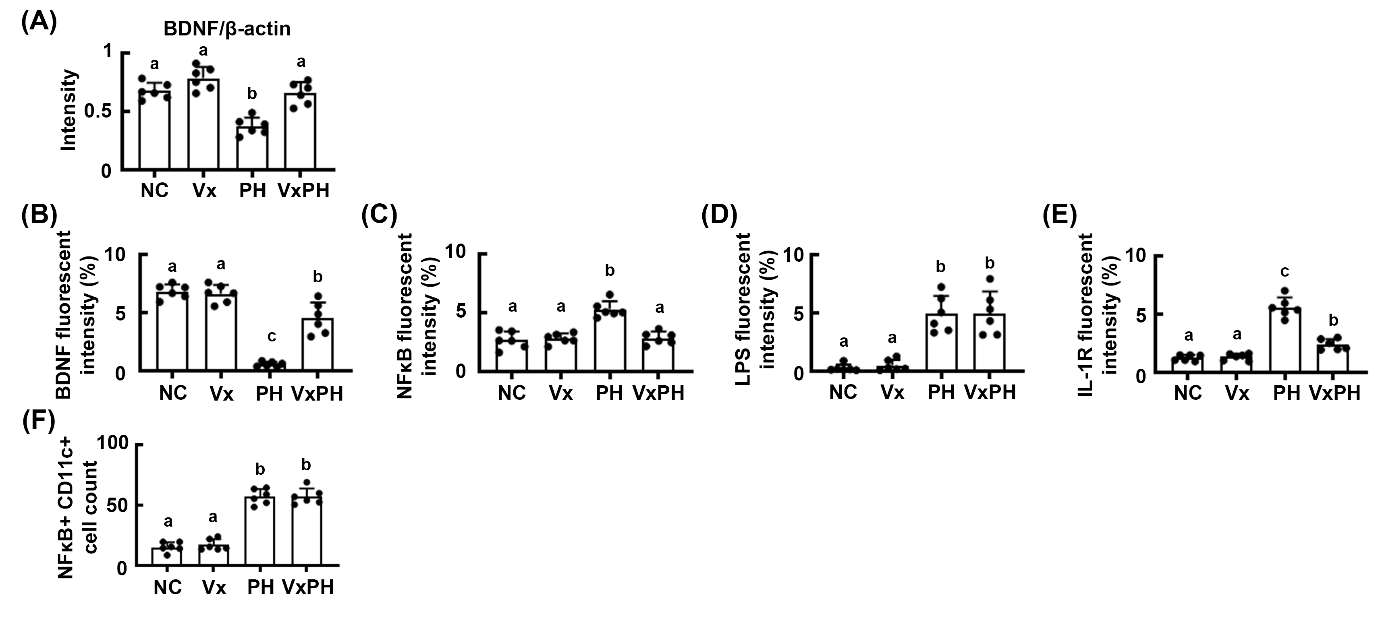


**Figure S9**. Intensities of Figure 5 immunoblotting and confocal microscope data. (A) Intensity of Figure 5B. (B) Intensity of Figure 5C. (C) Intensity of Figure 5D. (D) Intensity of Figure 5E. (E) Intensity of Figure 5F. (F) Intensity of Figure 5L. Data values were indicated as mean ± SD (n = 6). Means with same letters are not significantly different (p < 0.05). A-G, one-way ANOVA with post-hoc Bonferroni's multiple comparisons test.


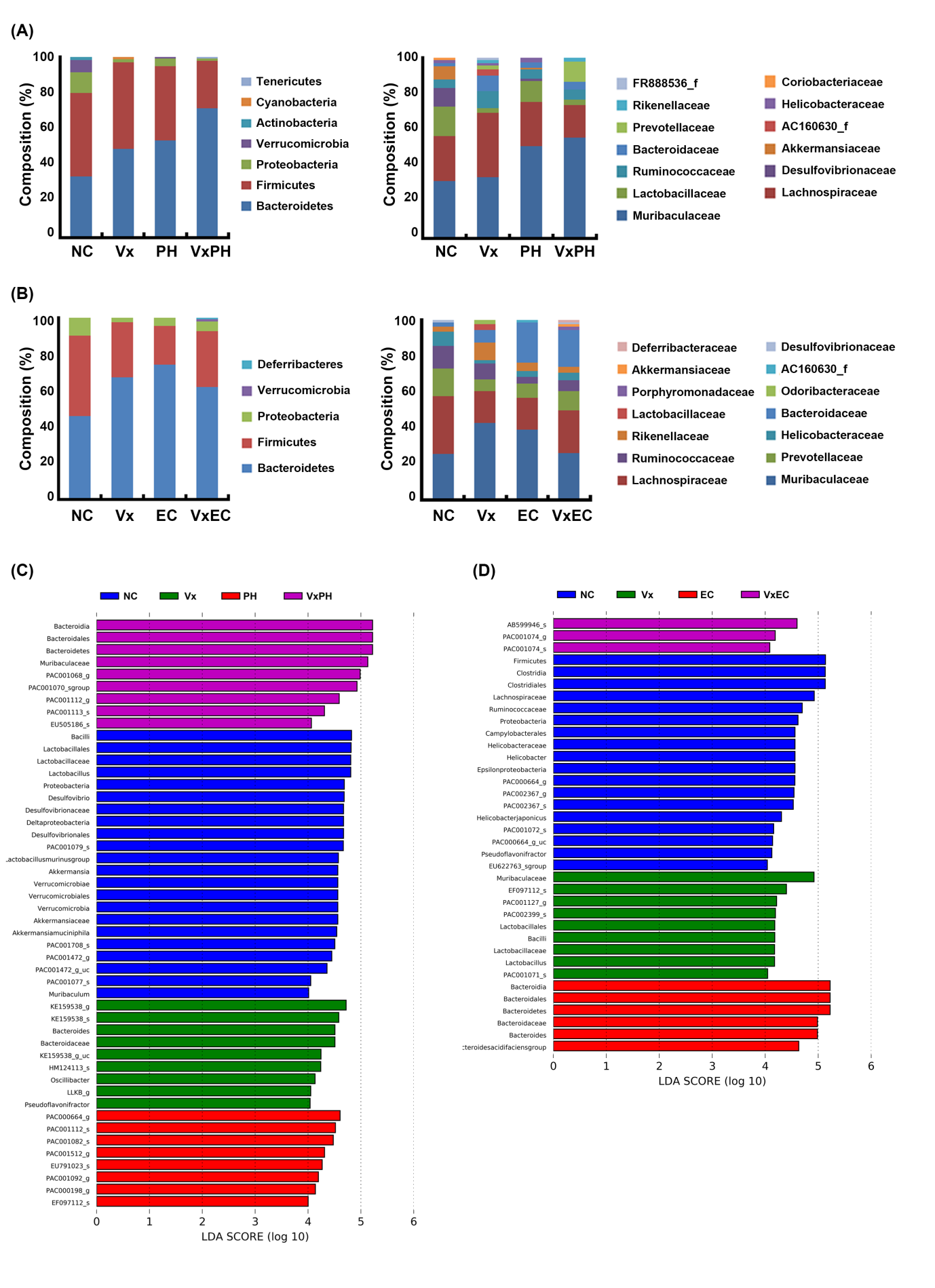


**Figure S10.** Effects of *Paenalcaligenes hominis* and *Escherichia coli* on the gut microbiota composition in mice with or without vagotomy. Effects of *Paenalcaligenes hominis* (A) and *Escherichia coli* (B) in phylum and family levels of fecal bacteria. Effects of *Paenalcaligenes hominis* (C) and *Escherichia coli* (D) on the Linear Discriminant Analysis (LDA) score of fecal bacteria. Fecal microbiota strains were analyzed to the LDA along with effect size measurement (LEfSE) in Galaxy (http://huttenhower.sph.harvard.edu/galaxy/). The threshold logarithmic score set at 4.0 and ranked. Bacterial strains were described based on 16SrRNA sequencing data. *Escherichia coli* (EC, 1×10^7^ CFU/mouse/day) and *Paenalcaligenes hominis* (PH, 1×10^7^ CFU/mouse/day) were orally gavaged for 5 days in mice with or without vagotomy. Control mice with (Vx) and without vagotomy (NC) were treated with vehicle (saline1% glucose) instead of bacterial suspension. Data values were indicated as mean ± SD (n = 6).


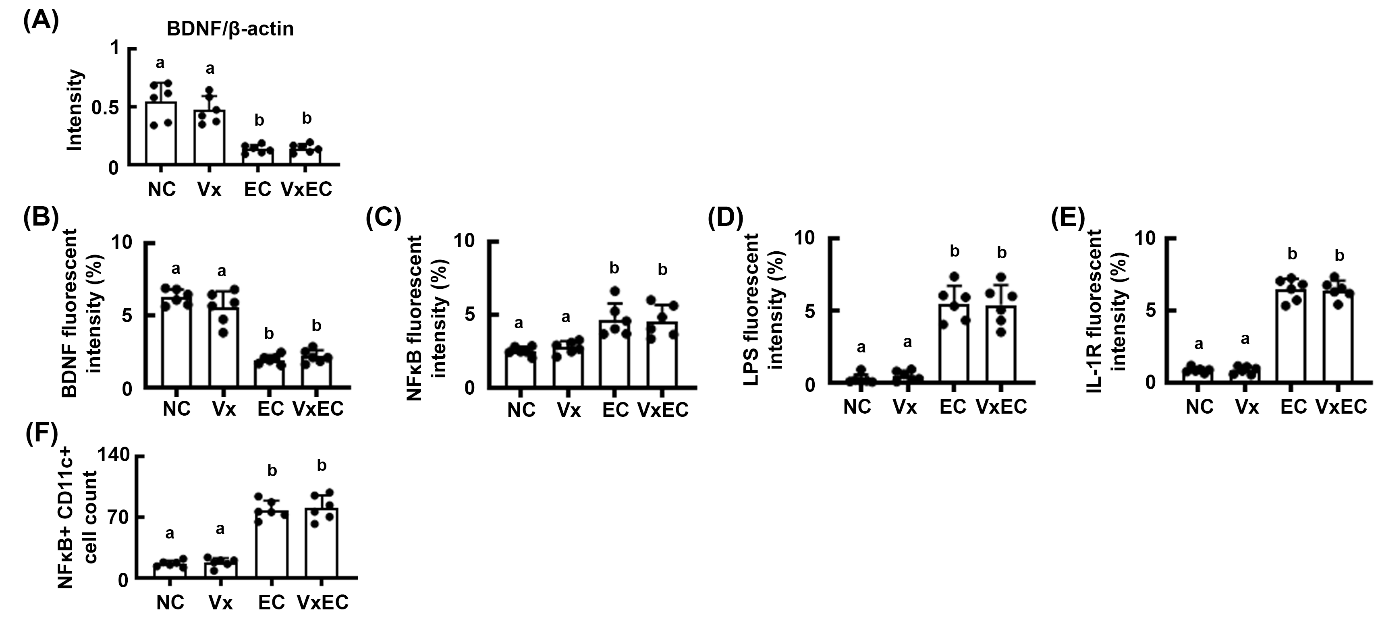


**Figure S11**. Intensities of Figure 6 immunoblotting and confocal microscope data. (A) Intensity of Figure 5B. (B) Intensity of Figure 5C. (C) Intensity of Figure 5D. (D) Intensity of Figure 5E. (E) Intensity of Figure 5F. (F) Intensity of Figure 2L. Data values were indicated as mean ± SD (n = 6). Means with same letters are not significantly different (p < 0.05). A-G, one-way ANOVA with post-hoc Bonferroni's multiple comparisons test.


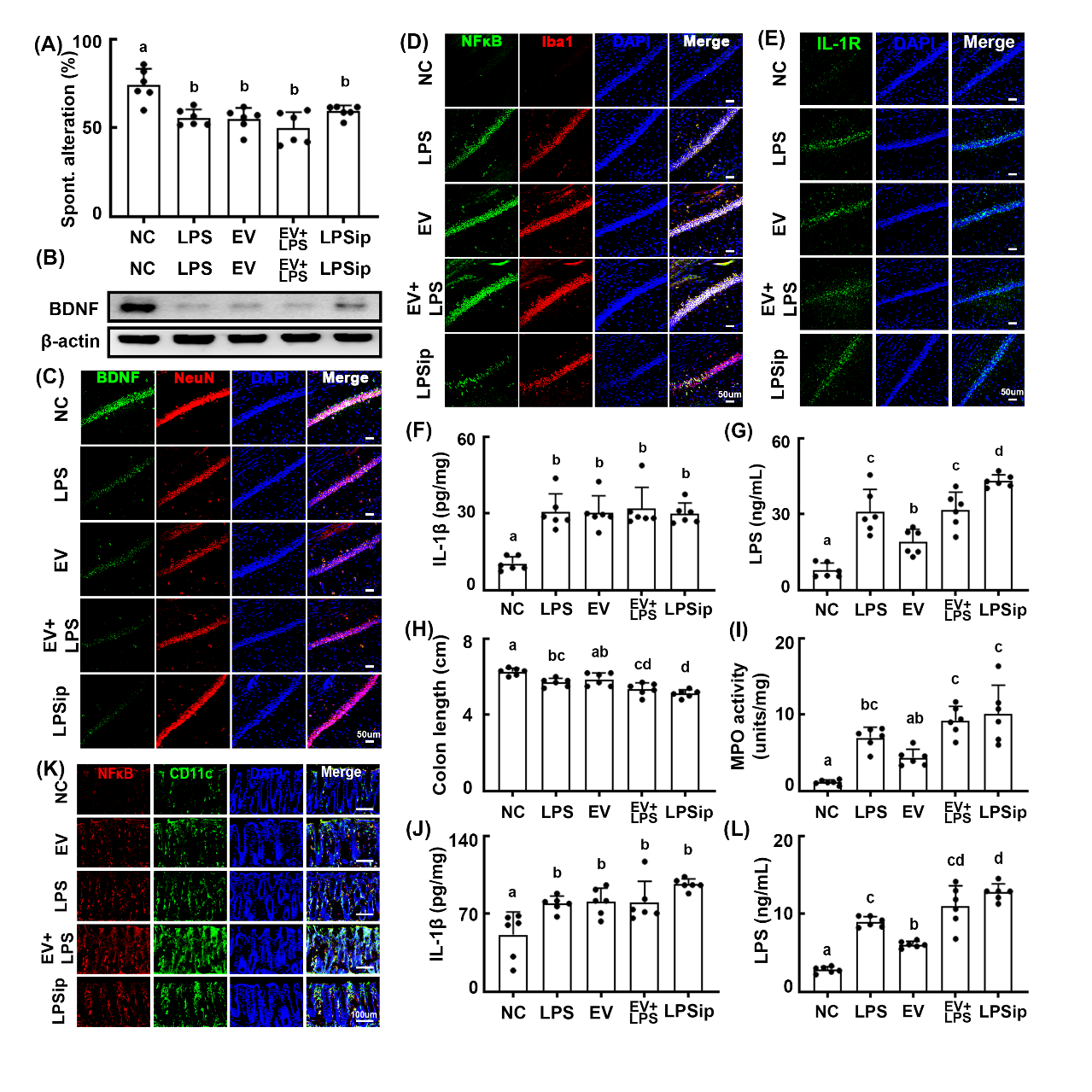


**Figure S12**. Effects of *Paenalcaligenes hominis* extracellular vesicles (EVs) and/or lipopolysaccharide (LPS) on the occurrence of cognitive impairment and colitis in mice with or without vagotomy. (A) Effects on the occurrence of cognitive impairment in Y-maze task. (B) Effects on the BDNF expression in the hippocampus. Effects on the infiltration of BDNF^+^/NeuN^+^ (C), NF-κB^+^/Iba1^+^ (D), and IL-1R^+^ cells (E) into the hippocampus. (F) Effects on the IL-1β expression in the hippocampus, assessed by ELISA. (G) Effects on the endotoxin levels in the blood, assessed by LAL assay kit. Effects on the colon length (H), myeloperoxidase (MPO) activity (I), IL-1β expression (J), and NF-κB^+^/CD11c^+^ cell population (K) in the colon. (L) Effects on the endotoxin levels in the feces. EV, LPS, and their mixture were orally gavaged (EV, LPS, and EV+LPS) or intraperitoneally injected (LPSip, treated with LPS) once a day for 5 days in mice. Control mice with (Vx) and without vagotomy (NC) were treated with vehicle (saline) instead of bacterial suspension. Data values were indicated as mean ± SD (n = 6). Means with same letters are not significantly different (p < 0.05). Data values were indicated as mean ± SD (n = 6). Means with same letters are not significantly different (p < 0.05). A,F-J, and L, one-way ANOVA with post-hoc Bonferroni's multiple comparisons test.


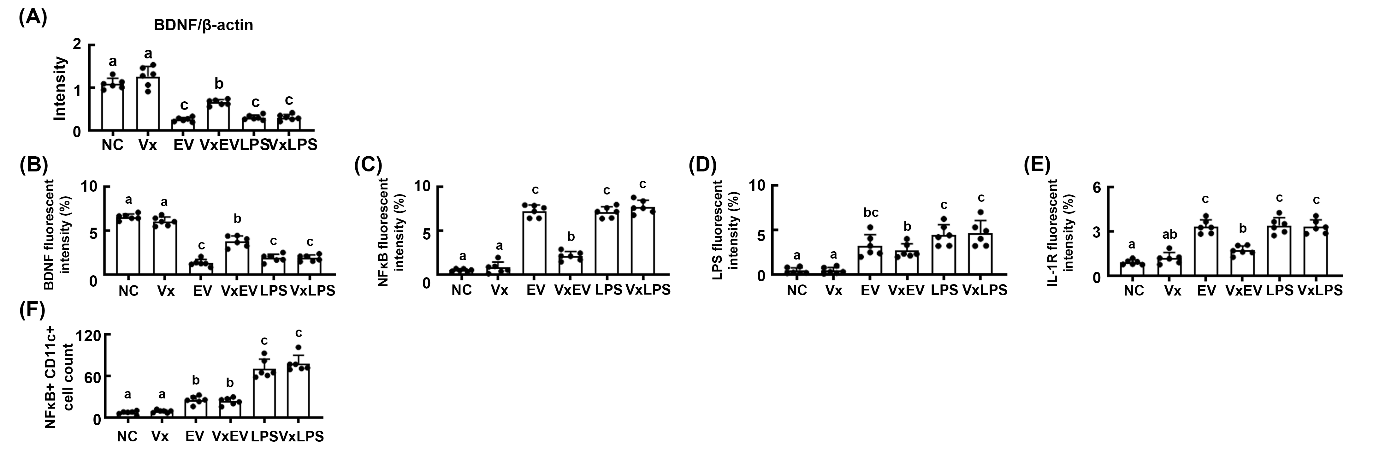


**Figure S13**. Intensities of Figure 7 immunoblotting and confocal microscope data. (A) Intensity of Figure 6B. (B) Intensity of Figure 6C. (C) Intensity of Figure 6D. (D) Intensity of Figure 6E. (E) Intensity of Figure 6F. (F) Intensity of Figure 6L. Data values were indicated as mean ± SD (n = 6). Means with same letters are not significantly different (p < 0.05). A-F, one-way ANOVA with post-hoc Bonferroni's multiple comparisons test.


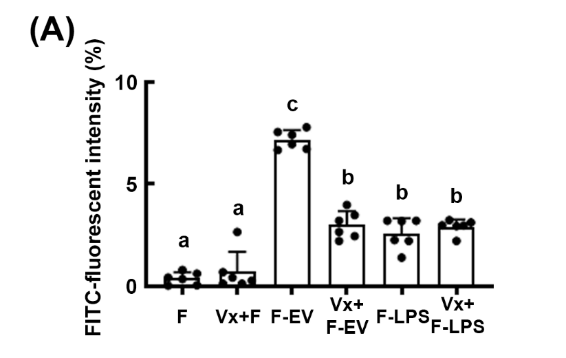


Figure S14. Intensities of Figure 8A confocal microscope data. Data values were indicated as mean ± SD (n = 6). Means with same letters are not significantly different (p < 0.05). Significance was analyzed using one-way ANOVA with post-hoc Bonferroni's multiple comparisons test.





**Figure S15**. *Paenalcaligenes hominis* and *Escherichia coli* on the expression of GABA and NMDA receptors in the hippocampus. *Paenalcaligenes hominis* (PH, 1×10^7^ CFU/mouse/day) and *Escherichia coli* (EC, 1×10^7^ CFU/mouse/day) were orally gavaged for 5 days. Control mice (NC) were treated with vehicle (saline) instead of bacterial suspension. Data values were indicated as mean ± SD (n = 6). Means with same letters are not significantly different (p < 0.05). Data values were indicated as mean ± SD (n = 6). Means with same letters are not significantly different (p < 0.05). A-D, one-way ANOVA with post-hoc Bonferroni's multiple comparisons test.


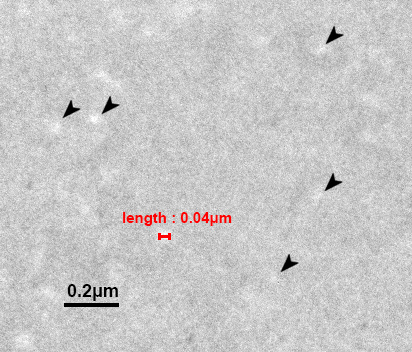


**Figure S16**. Transmission electron microscope image of *Paennalcaligenes hominis* (PH) extracellular vesicle (EV).


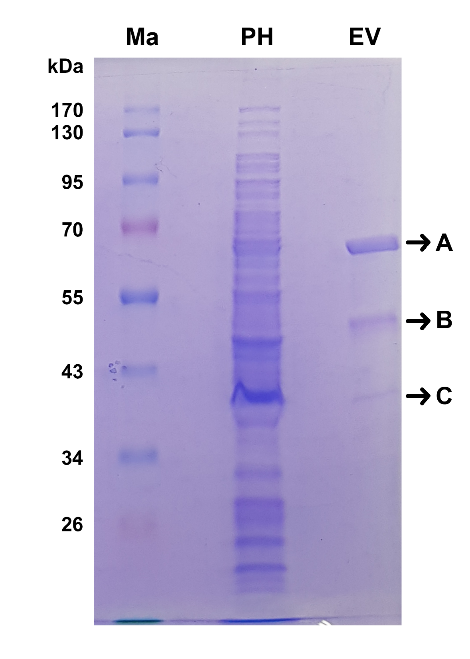


**Figure S17**. Sodium-polyacrylamide gel electrophoresis of intact PH and EV (A, B, and C, shown in Table S1).


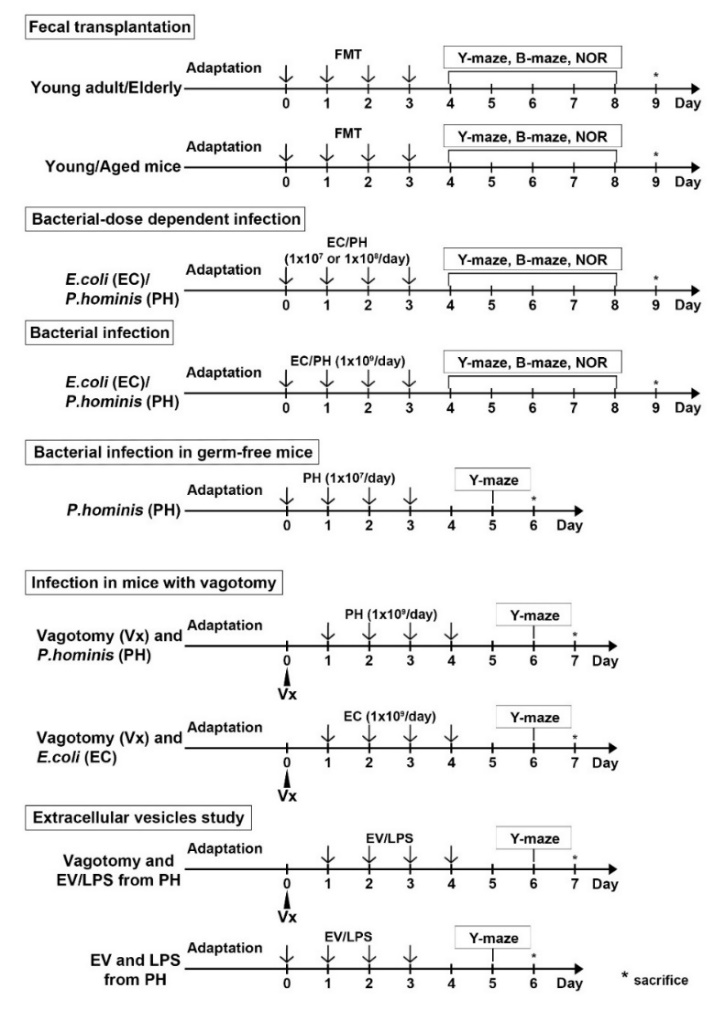


**Figure S18**. Protocols of in vivo experiments.





**Figure S19**. Accumulated effects of *Paenalcaligenes hominis* with or without vagotomy and *Escherichia coli* with or without vagotomy on the occurrence of cognitive impairment in the Y-maze task. NC, saline in mice (n=24); PH, *Paenalcaligenes hominis* in mice without vagotomy (n=24); VxPH, *Paenalcaligenes hominis* in mice with vagotomy (n=13); EC, *Escherichia coli* in mice without vagotomy (n=12); VxEC, *Escherichia coli* in mice with vagotomy (n=11); EV, extracellular vesicle (EV) in mice without vagotomy (n=20); VxEV, EV in mice with vagotomy (n=8); LPS, *Paenalcaligenes hominis* lipopolysaccharide (LPS) in mice without vagotomy (n=12); VxLPS, *Paenalcaligenes hominis* LPS in mice with vagotomy (n=6); EV+LPS, EV and LPS of *Paenalcaligenes hominis* ; and LPSip, interaperitoneally injected LPS. Test agents were orally gavaged in mice of all groups except LPSip group. Means with same letters are not significantly different (p < 0.05). Significance was analyzed using one-way ANOVA with post-hoc Bonferroni's multiple comparisons test.

**Table S1**. LC-MS-MS data of EV A, B, and C proteins

|  | Protein | Monoisotopic mass (M_r_) | Calculated pI | Matched peptide number | Protein sequence coverage (%) |
| --- | --- | --- | --- | --- | --- |
| A | hypothetical protein [Paenalcaligenes hominis] | 56576 | 4.29 | 18 | 17 |
| B | FliC/FljB family flagellin [Paenalcaligenes hominis] | 40459 | 5.27 | 15 | 17 |
| C | hypothetical protein [Paenalcaligenes hominis] | 56576 | 4.29 | 4 | 5 |

**Table S2.** Clinical characteristics of study participants

|  | Age (Y) (gender) | Body mass index^1)^ | Psychiatric disorders  (anxiety, depression, dementia) | Medicines within 3 months^2)^ |
| --- | --- | --- | --- | --- |
| Healthy young adult | Ave 20±3.6^3)^  22 (F)  23 (M)  15 (M)  20 (F) | 16.4  24.8  27.5  19.3 | No | No |
| Healthy elderly individual | Ave 62±1.8  60 (F)  64 (F)  61 (F)  62 (F) | 21.7  23.1  23.5  24.3 | No | No |

^1)^ *Body mass index (BMI) BMI is defined as the body mass divided by the square of the body height, and is universally expressed in units of kg/m^2^, resulting from mass in kilograms and height in metres.

^2)^ medications that can affect the gut microbiota, such as painkillers, anti-inflammatory drugs, and antibiotics for > 3 months. Acute gastrointestinal symptoms such as diarrhea and abdominal pain within the last week.

^3)^ Indicated as mean ± SD

**Table S3**. Primers for the qPCR of *Escherichia coli* and *Paenalcaligenes hominis*

|  | Primer | |
| --- | --- | --- |
|  | Forward | Reverse |
| *E.coli*^2^ | 5'-CAGCCACACTGGAACTGAG A-3' | 5'-GTTAGCCGGTGCTTCTTCT G-3' |
| *P. hominis* | 5'-AAAACCTTACCTACGCTTGA C-3' | 5'-ACCCAACATCTCACGACAC-3' |
| 16s rRNA | 5'-TCGTCGGCAGCGTCAGATGT  GTATAAGAGACAGGTGCCAGCMGCCGCGGTAA-3' | 5'-GTCTCGTGGGCTCGGAGAT GTGTATAAGAGACAGGGACTACHV GGGTWTCTAAT-3' |

**Table S4**. Primers for qPCR

|  | Primer [3-6] | |
| --- | --- | --- |
|  | Forward | Reverse |
| GABA_A_α1 | 5'-CCCGTTCAGTGGTTGTAGCA-3' | 5'-CTCTGTTGAGCCAGAAGGAG A C-3' |
| GABA_A_α2 | 5'-TTACAGTCCAAGCCGAATGTC C C-3' | 5'-ACTTCTGAGGTTGTGTAAGC GT AGC-3' |
| GABA_B_1b | 5'-CTCTTCTGCTGGTGATGGC-3' | 5'-TACTGCACGCCGTTCTGAG-3' |
| NMDA | 5'-ATTCATGCAGCCCTTTCAGA-3' | 5'-CCTTCCCCAATGCCAGAGT-3' |
| GAPDH | 5'-TGCAGTGGCAAAGTGGAGA T-3' | 5'-TTTGCCGTGAGTGGAGTCA TA-3' |

**Table S5**. P values of experimental data

| Figure 1. Human FMT  (A, B, C, I) Kruskal-Wallis test with Dunn's post-hoc test for non-parametric analysis.  (H, J, K) One-way ANOVA with post-hoc Bonferroni's multiple comparisons test. | | | | |
| --- | --- | --- | --- | --- |
|  | | NC vs. YF | NC vs. AF | YF vs. AF |
| (A) Spont. Alteration (%)  KW=11.83, p=0.0002 | | p>0.9999 | P=0.0035 | P=0.0280 |
| (B) Recog. Index (%)  KW=10.19, p=0.0020 | | p>0.9999 | p=0.0242 | p=0.0125 |
| (C)  Latency time (s) | 1D  KW=1.086, p>0.9999 | P>0.9999 | P>0.9999 | P>0.9999 |
|  | 2D  KW=1.940, p=0.4027 | P>0.9999 | P=0.5373 | P=0.9666 |
|  | 3D  KW=0.7257, p=0.7287 | P>0.9999 | P>0.9999 | P>0.9999 |
|  | 4D  KW=9.471, p=0.0021 | P>0.9999 | P=0.0321 | P=0.0171 |
| (H) IL-1β (pg/mg)  F=41.27, p<0.0001 | | P=0.0620 | P<0.0001 | p<0.0001 |
| (I) Colon length (cm)  KW=10.81, p=0.0010 | | P>0.9999 | p=0.0069 | p=0.0290 |
| (J) MPO activity (μunit/mg)  F=7.421, p=0.0057 | | P>0.9999 | p=0.0079 | P=0.0276 |
| (K) IL-1β (pg/mg)  F=15.94, p=0.0002 | | p=0.5691 | p=0.0002 | P=0.0031 |

| Figure 2.Bacterial infection in germ-free mouse  (A, G, H, I) Two tailed Mann-Whitney U test for non-parametric analysis.  (J) One tailed Mann-Whitney U test for non-parametric analysis. | | | | |
| --- | --- | --- | --- | --- |
|  | U statistic | Sum of ranks | | P value |
|  |  | NC | PH |  |
| (A) Spont. Alteration (%) | 0 | 26 | 10 | p=0.0286 |
| (G) IL-1β (pg/mg) | 0 | 10 | 26 | P=0.0286 |
| (H) Colon length (cm) | 0 | 26 | 10 | P=0.0286 |
| (I) MPO activity (μunit/mg) | 0 | 10 | 26 | P=0.0286 |
| (J) IL-1β (pg/mg) | 1 | 11 | 25 | P=0.0286 |

| Figure 3. Bacterial infection in conventional mouse  (A, B, C, K, L, M, N) Kruskal-Wallis ANOVA test with Dunn's post-hoc test for non-parametric analysis  (J, P) One-way ANOVA with post-hoc Bonferroni's multiple comparisons test | | | | |
| --- | --- | --- | --- | --- |
|  | | P value | | |
|  |  | NC vs. EC | NC vs. PH | EC vs. PH |
| (A) Spont. Alteration (%)  KW=11.42, p=0.0005 | | P=0.0035 | p=0.0447 | >0.9999 |
| (B) Recog. index (%)  KW=11.25, p=0.0006 | | p=0.0385 | P=0.0042 | p>0.9999 |
| (C)  Latency time (s) | 1D  KW=2, p>0.9999 | P=0.6620 | P=0.6620 | p>0.9999 |
|  | 2D  KW=7.220, p=0.0193 | p>0.9999 | P=0.0267 | P=0.1980 |
|  | 3D  KW=6.346, p=0.0324 | P=0.4708 | P=0.0360 | P=0.8179 |
|  | 4D  KW=9.557, p=0.0020 | P=0.0348 | P=0.0149 | p>0.9999 |
| (J) IL-1β (pg/mg)  F=49.89, p<0.0001 | | P<0.0001 | P<0.0001 | P=0.0346 |
| (K) LPS (ng/mL)  KW=11.76, p=0.0003 | | P=0.0259 | P=0.0038 | p>0.9999 |
| (L) Colon length (cm)  KW=10.24, p=0.0020 | | P= 0.0168 | P=0.0168 | p>0.9999 |
| (M) MPO activity (μunit/mg)  KW=11.42, p=0.0005 | | P=0.0148 | P=0.0074 | p>0.9999 |
| (N) IL-1β (pg/mg)  KW=11.42, p=0.0005 | | P=0.0148 | P=0.0074 | p>0.9999 |
| (P) LPS (ng/mg)  F=25.47, p<0.0001 | | p<0.0001 | P=0.0215 | P=0.0034 |

| Figure 4. Mating  (A, B, C) One-way ANOVA with post-hoc Bonferroni's multiple comparisons test.  (D) Two tailed Mann-Whitney U test for non-parametric analysis. | | | | | | | | | |
| --- | --- | --- | --- | --- | --- | --- | --- | --- | --- |
| (A) | | | | | | | | | |
| (a) male *E.coli*  (fold change)  F=17.61, p<0.0001 | | NC vs. 30d-NC | | p>0.9999 | | 30d-NC vs. EC | | | P<0.0001 |
|  |  | NC vs. EC | | P<0.0001 | | 30d-NC vs. 30d-EC | | | p>0.9999 |
|  |  | NC vs. 30d-EC | | p>0.9999 | | EC vs. 30d-EC | | | P<0.0001 |
| (b) female *E.coli*  (fold change)  F= 57.92, p<0.0001 | | NC vs. 30d-NC | | p>0.9999 | | 30d-NC vs. EC | | | P<0.0001 |
|  |  | NC vs. EC | | P<0.0001 | | 30d-NC vs. 30d-EC | | | p>0.9999 |
|  |  | NC vs. 30d-EC | | p>0.9999 | | EC vs. 30d-EC | | | P<0.0001 |
| (c) male *P.hominis*  (fold change)  F=12.60, p<0.0001 | | NC vs. 30d-NC | | p>0.9999 | | 30d-NC vs. PH | | | P=0.0003 |
|  |  | NC vs. PH | | P=0.0003 | | 30d-NC vs. 30d-PH | | | p>0.9999 |
|  |  | NC vs. 30d-PH | | p>0.9999 | | PH vs. 30d-PH | | | P=0.0005 |
| (d) female *P.hominis*  (fold change)  F=62.71, p<0.0001 | | NC vs. 30d-NC | | p>0.9999 | | 30d-NC vs. PH | | | p<0.0001 |
|  |  | NC vs. PH | | p<0.0001 | | 30d-NC vs. 30d-PH | | | p>0.9999 |
|  |  | NC vs. 30d-PH | | p>0.9999 | | PH vs. 30d-PH | | | p<0.0001 |
| (B) | | | | | | | | | |
| (a)  Spont. Alteration (%)  F=9.945, p<0.0001 | NC vs. 30d-NC | | | p>0.9999 | | 30d-NC vs. 30d-PH | | | P=0.0390 |
|  | NC vs. EC | | | P=0.0005 | | EC vs. 30d-EC | | | P=0.0105 |
|  | NC vs. 30d-EC | | | p>0.9999 | | EC vs. PH | | | p>0.9999 |
|  | NC vs. PH | | | P=0.0006 | | EC vs. 30d-PH | | | p>0.9999 |
|  | NC vs. 30d-PH | | | P=0.0140 | | 30d-EC vs. PH | | | P=0.0124 |
|  | 30d-NC vs. EC | | | P=0.0016 | | 30d-EC vs. 30d-PH | | | P=0.2138 |
|  | 30d-NC vs. 30d-EC | | | p>0.9999 | | PH vs. 30d-PH | | | p>0.9999 |
|  | 30d-NC vs. PH | | | P=0.0019 | |  | | | |
| (b) IL-1β (pg/mg)  F=109.3, p<0.0001 | 1d-NC vs. 30d-NC | | | p>0.9999 | | 30d-NC vs. 30d-PH | | | p>0.9999 |
|  | 1d-NC vs. 1d-EC | | | p<0.0001 | | 1d-EC vs. 30d-EC | | | p<0.0001 |
|  | 1d-NC vs. 30d-EC | | | p>0.9999 | | 1d-EC vs. 1d-PH | | | P=0.0031 |
|  | 1d-NC vs. 1d-PH | | | p<0.0001 | | 1d-EC vs. 30d-PH | | | p<0.0001 |
|  | 1d-NC vs. 30d-PH | | | p>0.9999 | | 30d-EC vs. 1d-PH | | | p<0.0001 |
|  | 30d-NC vs. 1d-EC | | | p<0.0001 | | 30d-EC vs. 30d-PH | | | p>0.9999 |
|  | 30d-NC vs. 30d-EC | | | p>0.9999 | | 1d-PH vs. 30d-PH | | | p<0.0001 |
|  | 30d-NC vs. 1d-PH | | | p<0.0001 | |  | | | |
| (C) | | | | | | | | | |
| (a) MPO activity (μunit/mg)  F=36.85  p<0.0001 | 1d-NC vs. 30d-NC | | | p>0.9999 | | 30d-NC vs. 30d-PH | | | p>0.9999 |
|  | 1d-NC vs. 1d-EC | | | p<0.0001 | | 1d-EC vs. 30d-EC | | | p<0.0001 |
|  | 1d-NC vs. 30d-EC | | | p>0.9999 | | 1d-EC vs. 1d-PH | | | p>0.9999 |
|  | 1d-NC vs. 1d-PH | | | p<0.0001 | | 1d-EC vs. 30d-PH | | | p<0.0001 |
|  | 1d-NC vs. 30d-PH | | | p>0.9999 | | 30d-EC vs. 1d-PH | | | p<0.0001 |
|  | 30d-NC vs. 1d-EC | | | p<0.0001 | | 30d-EC vs. 30d-PH | | | p>0.9999 |
|  | 30d-NC vs. 30d-EC | | | p>0.9999 | | 1d-PH vs. 30d-PH | | | p<0.0001 |
|  | 30d-NC vs. 1d-PH | | | p<0.0001 | |  | | | |
| (b) IL-1β (pg/mg)  F=81.24, p<0.0001 | 1d-NC vs. 30d-NC | | | p>0.9999 | | 30d-NC vs. 30d-PH | | | p>0.9999 |
|  | 1d-NC vs. 1d-EC | | | p<0.0001 | | 1d-EC vs. 30d-EC | | | p<0.0001 |
|  | 1d-NC vs. 30d-EC | | | p>0.9999 | | 1d-EC vs. 1d-PH | | | p>0.9999 |
|  | 1d-NC vs. 1d-PH | | | p<0.0001 | | 1d-EC vs. 30d-PH | | | p<0.0001 |
|  | 1d-NC vs. 30d-PH | | | p>0.9999 | | 30d-EC vs. 1d-PH | | | p<0.0001 |
|  | 30d-NC vs. 1d-EC | | | p<0.0001 | | 30d-EC vs. 30d-PH | | | p>0.9999 |
|  | 30d-NC vs. 30d-EC | | | p>0.9999 | | 1d-PH vs. 30d-PH | | | p<0.0001 |
|  | 30d-NC vs. 1d-PH | | | p<0.0001 | |  | | | |
| (D) | | | | | | | | | |
|  | | | U statistic | | Sum of ranks | | | P value | |
| (a) *E.coli* (fold change) from pup | | | 15 | | NC | | 36 | p>0.9999 | |
|  |  |  |  |  | EC | | 30 |  |  |
| (b) *P.hominis* (fold change) from pup | | | 9 | | NC | | 30 | P=0.1797 | |
|  |  |  |  |  | PH | | 48 |  |  |

| Figure 5. Vagotomy and PH  (A, G, H, I, J, K, O) One-way ANOVA with post-hoc Bonferroni's multiple comparisons test.  (P) One-way ANOVA with post-hoc Holm-Sidak's multiple comparisons test. | | | | |
| --- | --- | --- | --- | --- |
| (A) Spont. alteration (%)  F=13.63, p<0.0001 | NC vs. Vx | p>0.9999 | Vx vs. PH | P=0.0003 |
|  | NC vs. PH | p<0.0001 | Vx vs. VxPH | P=0.6758 |
|  | NC vs. VxPH | P=0.1650 | PH vs. VxPH | P=0.0142 |
| (G) IL-1β (pg/mg)  F=24.92, p<0.0001 | NC vs. Vx | p>0.9999 | Vx vs. PH | p<0.0001 |
|  | NC vs. PH | p<0.0001 | Vx vs. VxPH | P=0.1696 |
|  | NC vs. VxPH | P=0.0078 | PH vs. VxPH | P=0.0020 |
| (H) LPS (ng/mL)  F= 9.339, p=0.0005 | NC vs. Vx | p>0.9999 | Vx vs. PH | P=0.0089 |
|  | NC vs. PH | P=0.0034 | Vx vs. VxPH | P=0.0188 |
|  | NC vs. VxPH | P=0.0073 | PH vs. VxPH | p>0.9999 |
| (I) Colon length (cm)  F=21.47, p<0.0001 | NC vs. Vx | p>0.9999 | Vx vs. PH | p<0.0001 |
|  | NC vs. PH | p<0.0001 | Vx vs. VxPH | P=0.0003 |
|  | NC vs. VxPH | P=0.0002 | PH vs. VxPH | p>0.9999 |
| (J) MPO activity (μunit/mg)  F=36.20, p<0.0001 | NC vs. Vx | p>0.9999 | Vx vs. PH | p<0.0001 |
|  | NC vs. PH | p<0.0001 | Vx vs. VxPH | p<0.0001 |
|  | NC vs. VxPH | p<0.0001 | PH vs. VxPH | p>0.9999 |
| (K) IL-1β (pg/mg)  F=17.31, p<0.0001 | NC vs. Vx | p>0.9999 | Vx vs. PH | P=0.0060 |
|  | NC vs. PH | P=0.0005 | Vx vs. VxPH | P=0.0004 |
|  | NC vs. VxPH | p<0.0001 | PH vs. VxPH | p>0.9999 |
| (O) OTUs  F=2.134, p=0.1278 | NC vs. Vx | p>0.9999 | Vx vs. PH | P=0.2525 |
|  | NC vs. PH | P=0.5704 | Vx vs. VxPH | p>0.9999 |
|  | NC vs. VxPH | p>0.9999 | PH vs. VxPH | P=0.2574 |
| (P) LPS (ng/mg)  F=6.088, p=0.0041 | NC vs. Vx | P=0.8832 | Vx vs. PH | P=0.0338 |
|  | NC vs. PH | P=0.0184 | Vx vs. VxPH | P=0.0472 |
|  | NC vs. VxPH | P=0.0291 | PH vs. VxPH | P=0.8832 |

| Figure 6. Vagotomy and EC  One-way ANOVA with post-hoc Bonferroni's multiple comparisons test. | | | | |
| --- | --- | --- | --- | --- |
| (A) Spont. alteration (%)  F=10.43, p=0.0002 | NC vs. Vx | p>0.9999 | Vx vs. EC | P=0.0025 |
|  | NC vs. EC | P=0.0012 | Vx vs. VxEC | P=0.0263 |
|  | NC vs. VxEC | P=0.0124 | EC vs. VxEC | p>0.9999 |
| (G) IL-1β (pg/mg)  F=55.58, p<0.0001 | NC vs. Vx | P=0.0841 | Vx vs. EC | p<0.0001 |
|  | NC vs. EC | p<0.0001 | Vx vs. VxEC | p<0.0001 |
|  | NC vs. VxEC | p<0.0001 | EC vs. VxEC | P=0.2715 |
| (H) LPS (ng/mL)  F=17.48, p<0.0001 | NC vs. Vx | p>0.9999 | Vx vs. EC | P=0.0005 |
|  | NC vs. EC | p<0.0001 | Vx vs. VxEC | P=0.0029 |
|  | NC vs. VxEC | P=0.0003 | EC vs. VxEC | p>0.9999 |
| (I) Colon length (cm)  F=12.51, p <0.0001 | NC vs. Vx | p>0.9999 | Vx vs. EC | P=0.0017 |
|  | NC vs. EC | P=0.0022 | Vx vs. VxEC | P=0.0017 |
|  | NC vs. VxEC | P=0.0022 | EC vs. VxEC | p>0.9999 |
| (J) MPO activity (μunit/mg)  F=45.71, p<0.0001 | NC vs. Vx | p>0.9999 | Vx vs. EC | p<0.0001 |
|  | NC vs. EC | p<0.0001 | Vx vs. VxEC | p<0.0001 |
|  | NC vs. VxEC | p<0.0001 | EC vs. VxEC | p>0.9999 |
| (K) IL-1β (pg/mg)  F=14.79, p<0.0001 | NC vs. Vx | P=0.6967 | Vx vs. EC | P=0.0040 |
|  | NC vs. EC | p<0.0001 | Vx vs. VxEC | P=0.0160 |
|  | NC vs. VxEC | P=0.0004 | EC vs. VxEC | p>0.9999 |
| (N) OTUs  F=0.2825, p=0.8374 | NC vs. Vx | p>0.9999 | Vx vs. EC | p>0.9999 |
|  | NC vs. EC | p>0.9999 | Vx vs. VxEC | p>0.9999 |
|  | NC vs. VxEC | p>0.9999 | EC vs. VxEC | p>0.9999 |
| (P) LPS (ng /mg)  F=14.79, p<0.0001 | NC vs. Vx | P=0.6967 | Vx vs. EC | P=0.0040 |
|  | NC vs. EC | p<0.0001 | Vx vs. VxEC | P=0.0160 |
|  | NC vs. VxEC | P=0.0004 | EC vs. VxEC | p>0.9999 |

| Figure 7. Vagotomy  One-way ANOVA with post-hoc Bonferroni's multiple comparisons test. | | | | |
| --- | --- | --- | --- | --- |
| (A) Spont. alteration (%)  F=16.51, p<0.0001 | NC vs. Vx | p>0.9999 | Vx vs. VxLPS | P=0.0002 |
|  | NC vs. EV | p<0.0001 | EV vs. VxEV | P=0.0265 |
|  | NC vs. VxEV | P=0.0116 | EV vs. LPS | P=0.6995 |
|  | NC vs. LPS | P=0.0003 | EV vs. VxLPS | p>0.9999 |
|  | NC vs. VxLPS | p<0.0001 | VxEV vs. LPS | p>0.9999 |
|  | Vx vs. EV | p<0.0001 | VxEV vs. VxLPS | P=0.5413 |
|  | Vx vs. VxEV | P=0.0853 | LPS vs. VxLPS | p>0.9999 |
|  | Vx vs. LPS | P=0.0023 |  |  |
| (G) IL-1β (pg/mg)  F=17.44, p<0.0001 | NC vs. Vx | p>0.9999 | Vx vs. VxLPS | p<0.0001 |
|  | NC vs. EV | p<0.0001 | EV vs. VxEV | P=0.0428 |
|  | NC vs. VxEV | P=0.1201 | EV vs. LPS | p>0.9999 |
|  | NC vs. LPS | p<0.0001 | EV vs. VxLPS | p>0.9999 |
|  | NC vs. VxLPS | p<0.0001 | VxEV vs. LPS | P=0.0476 |
|  | Vx vs. EV | p<0.0001 | VxEV vs. VxLPS | P=0.0296 |
|  | Vx vs. VxEV | P=0.2420 | LPS vs. VxLPS | p>0.9999 |
|  | Vx vs. LPS | p<0.0001 |  |  |
| (H) LPS (ng/mL)  F=15.77, p<0.0001 | NC vs. Vx | p>0.9999 | Vx vs. VxLPS | p<0.0001 |
|  | NC vs. EV | P=0.0045 | EV vs. VxEV | p>0.9999 |
|  | NC vs. VxEV | P=0.0029 | EV vs. LPS | P=0.7282 |
|  | NC vs. LPS | p<0.0001 | EV vs. VxLPS | P=0.0937 |
|  | NC vs. VxLPS | p<0.0001 | VxEV vs. LPS | p>0.9999 |
|  | Vx vs. EV | P=0.0415 | VxEV vs. VxLPS | P=0.1384 |
|  | Vx vs. VxEV | P=0.0274 | LPS vs. VxLPS | p>0.9999 |
|  | Vx vs. LPS | P=0.0001 |  |  |
| (I) Colon length (cm)  F=7.583, p=0.0001 | NC vs. Vx | p>0.9999 | Vx vs. VxLPS | P=0.0010 |
|  | NC vs. EV | P=0.5044 | EV vs. VxEV | p>0.9999 |
|  | NC vs. VxEV | P=0.3534 | EV vs. LPS | p>0.9999 |
|  | NC vs. LPS | P=0.0061 | EV vs. VxLPS | P=0.1677 |
|  | NC vs. VxLPS | P=0.0004 | VxEV vs. LPS | p>0.9999 |
|  | Vx vs. EV | P=0.9890 | VxEV vs. VxLPS | P=0.2448 |
|  | Vx vs. VxEV | P=0.7110 | LPS vs. VxLPS | p>0.9999 |
|  | Vx vs. LPS | P=0.0145 |  |  |
| (J) MPO activity (μunit/mg)  F=41.86, p<0.0001 | NC vs. Vx | P>0.9999 | Vx vs. VxLPS | p<0.0001 |
|  | NC vs. EV | p<0.0001 | EV vs. VxEV | p>0.9999 |
|  | NC vs. VxEV | p<0.0001 | EV vs. LPS | P=0.0082 |
|  | NC vs. LPS | p<0.0001 | EV vs. VxLPS | P=0.0013 |
|  | NC vs. VxLPS | p<0.0001 | VxEV vs. LPS | P=0.0196 |
|  | Vx vs. EV | p<0.0001 | VxEV vs. VxLPS | P=0.0032 |
|  | Vx vs. VxEV | p<0.0001 | LPS vs. VxLPS | p>0.9999 |
|  | Vx vs. LPS | p<0.0001 |  |  |
| (K) IL-1β (pg/mg)  F=14.09, p<0.0001 | NC vs. Vx | p>0.9999 | Vx vs. VxLPS | p<0.0001 |
|  | NC vs. EV | P=0.0006 | EV vs. VxEV | p>0.9999 |
|  | NC vs. VxEV | P=0.0004 | EV vs. LPS | p>0.9999 |
|  | NC vs. LPS | P=0.0016 | EV vs. VxLPS | p>0.9999 |
|  | NC vs. VxLPS | p<0.0001 | VxEV vs. LPS | p>0.9999 |
|  | Vx vs. EV | P=0.0018 | VxEV vs. VxLPS | p>0.9999 |
|  | Vx vs. VxEV | P=0.0012 | LPS vs. VxLPS | P=0.8371 |
|  | Vx vs. LPS | P=0.0052 |  |  |
| (M) LPS (ng/mg)  F=27.50, p<0.0001 | NC vs. Vx | p>0.9999 | Vx vs. VxLPS | p<0.0001 |
|  | NC vs. EV | P=0.0109 | EV vs. VxEV | p>0.9999 |
|  | NC vs. VxEV | P=0.0113 | EV vs. LPS | P=0.0007 |
|  | NC vs. LPS | p<0.0001 | EV vs. VxLPS | P<0.0001 |
|  | NC vs. VxLPS | p<0.0001 | VxEV vs. LPS | P=0.0007 |
|  | Vx vs. EV | p>0.9999 | VxEV vs. VxLPS | p<0.0001 |
|  | Vx vs. VxEV | p>0.9999 | LPS vs. VxLPS | p>0.9999 |
|  | Vx vs. LPS | p<0.0001 |  | p<0.0001 |

| Figure 8. Vagotomy  One-way ANOVA with post-hoc Bonferroni's multiple comparisons test. | | | | |
| --- | --- | --- | --- | --- |
| (B) 16s DNA (fold change)  F=8.272, p<0.0001 | NC vs. Vx | p>0.9999 | Vx vs. VxEV | P=0.1212 |
|  | NC vs. PH | P=0.0063 | PH vs. VxPH | P=0.7798 |
|  | NC vs. VxPH | P=0.9229 | PH vs. EV | p>0.9999 |
|  | NC vs. EV | P=0.0017 | PH vs. VxEV | p>0.9999 |
|  | NC vs. VxEV | P=0.5120 | VxPH vs. EV | P=0.2786 |
|  | Vx vs. PH | P=0.0011 | VxPH vs. VxEV | p>0.9999 |
|  | Vx vs. VxPH | P=0.2362 | EV vs. VxEV | P=0.5211 |
|  | Vx vs. EV | P=0.0003 |  |  |

P values of Supplementary information data

| Supplemental Figure S1. The number of fecal bacterial colonies  two tailed Mann-Whitney U test. | | | | | |
| --- | --- | --- | --- | --- | --- |
|  | | U statics | Sum of ranks | | P value |
| (A) Ym vs Am | BL Agar | 0 | Ym | 57 | P=0.0022 |
|  |  |  | Am | 21 |  |
|  | DHL Agar | 4 | Ym | 25 | P=0.0260 |
|  |  |  | Am | 53 |  |
| (B) Yh vs Ah | BL Agar | 0 | Yh | 26 | P=0.0286 |
|  |  |  | Ah | 10 |  |
|  | DHL Agar | 0 | Yh | 10 | P=0.0286 |
|  |  |  | Ah | 26 |  |
| (C) Ym vs Am | Paenalcaligenes hominis  (fold change) | 0 | Ym | 21 | P=0.0022 |
|  |  |  | Am | 57 |  |
|  | Escherichia coli  (fold change) | 3 | Ym | 24 | P=0.0152 |
|  |  |  | Am | 54 |  |
| (D) Yh vs Ah | Paenalcaligenes hominis  (fold change) | 0 | Yh | 10 | P=0.0286 |
|  |  |  | Ah | 26 |  |
|  | Escherichia coli  (fold change) | 2 | Yh | 12 | P=0.1143 |
|  |  |  | Ah | 24 |  |

| Supplemental Figure S2. Effects of young and aged mouse fecal transplantations  two tailed Mann-Whitney U test | | | | | | |
| --- | --- | --- | --- | --- | --- | --- |
|  | | | U statics | Sum of ranks | | P value |
|  |  |  |  | YF | AF |  |
| (A) Spont. Alteration (%) | | | 2 | 23 | 55 | p=0.0087 |
| (B) Recog. Index (%) | | | 2 | 23 | 55 | p=0.0087 |
| (C) Latency time (s) | | 1D | 10 | 25 | 30 | p>0.9999 |
|  |  | 2D | 2.500 | 17.50 | 37.50 | P=0.0397 |
|  |  | 3D | 0 | 15 | 40 | P=0.0079 |
|  |  | 4D | 0 | 15 | 40 | P=0.0079 |
| (D) Western blot | BDNF/β-actin | | 3 | 54 | 24 | P=0.0152 |
|  | p-p65/p-65 | | 3 | 24 | 54 | P=0.0152 |
|  | P16/β-actin | | 0 | 21 | 57 | P=0.0022 |
| (E) BDNF fluorescent intensity (%) | | | 0 | 57 | 21 | P=0.0022 |
| (F) NFκB fluorescent intensity (%) | | | 0 | 21 | 57 | P=0.0022 |
| (G) LPS fluorescent intensity (%) | | | 0 | 21 | 57 | P=0.0022 |
| (H) IL-1β (pg/mg) | | | 2 | 23 | 55 | p=0.0087 |
| (I) Colon length (cm) | | | 3 | 54 | 24 | p=0.0173 |
| (J) MPO activity (μunit/mg) | | | 2 | 23 | 55 | p=0.0087 |
| (K) IL-1β (pg/mg) | | | 0 | 21 | 57 | P=0.0022 |

| Supplemental Figure S3. Figure 1 intensity  one-way ANOVA with post-hoc Bonferroni's multiple comparisons test | | | | |
| --- | --- | --- | --- | --- |
|  | | NC vs. YF | NC vs. AF | YF vs. AF |
| (A) BDNF fluorescent intensity (%)  F=49.39, p<0.0001 | | p>0.9999 | p<0.0001 | p<0.0001 |
| (B) NFκB fluorescent intensity (%)  F=140.9, p<0.0001 | | P=0.3800 | p<0.0001 | p<0.0001 |
| (C) LPS fluorescent intensity (%)  F=62.88, p<0.0001 | | p>0.9999 | p<0.0001 | p<0.0001 |
| (D) Western blot | BDNF/β-actin  F=12.26, p=0.0007 | P=0.3844 | P=0.0006 | P=0.0162 |
|  | p-p65/p-65  F=12.09, p=0.0007 | P>0.9999 | P=0.0032 | P=0.0014 |
|  | P16/β-actin  F=9.002, p=0.0027 | p>0.9999 | P=0.0057 | P=0.0082 |
| (E) Western blot | p-p65/p-65  F=8.324, p=0.0037 | p>0.9999 | P=0.0048 | P=0.0211 |
|  | P16/β-actin  F=23.68, p<0.0001 | P=0.1286 | p<0.0001 | P=0.0012 |
| NFκB+cd11c+ cell count  F=39.22, p<0.0001 | | p>0.9999 | p<0.0001 | P<0.0001 |

| Supplemental Figure S4.  (A, B) one-way ANOVA with post-hoc Bonferroni's multiple comparisons test.  (C, D) two tailed Mann-Whitney U test for non-parametric analysis. | | | | | | | | |
| --- | --- | --- | --- | --- | --- | --- | --- | --- |
| (A) | | | | | | | | |
| (a) Spont. Alteration (%)  F=4.821, p=0.0051 | NC vs. Yh | | P=0.0499 | | Yh vs. Ym | | P=0.9995 | |
|  | NC vs. Ah | | P=0.0092 | | Yh vs. Am | | P=0.9645 | |
|  | NC vs. Ym | | P=0.0499 | | Ah vs. Ym | | P=0.9645 | |
|  | NC vs. Am | | P=0.0092 | | Ah vs. Am | | P=0.9995 | |
|  | Yh vs. Ah | | P=0.9645 | | Ym vs. Am | | P=0.9645 | |
| (b) IL-1β (pg/mg)  F=25.43, p<0.0001 | NC vs. Yh | | P=0.0041 | | Yh vs. Ym | | p>0.9999 | |
|  | NC vs. Ah | | p<0.0001 | | Yh vs. Am | | P=0.0001 | |
|  | NC vs. Ym | | P=0.0045 | | Ah vs. Ym | | P=0.0725 | |
|  | NC vs. Am | | p<0.0001 | | Ah vs. Am | | P=0.1775 | |
|  | Yh vs. Ah | | P=0.0789 | | Ym vs. Am | | P=0.0001 | |
| (B) | | | | | | | | |
| (a) MPO activity (μunit/mg)  F=7.492, p= 0.0004 | NC vs. Yh | | P=0.0375 | | Yh vs. Ym | | p>0.9999 | |
|  | NC vs. Ah | | P=0.0048 | | Yh vs. Am | | P=0.7887 | |
|  | NC vs. Ym | | P=0.0041 | | Ah vs. Ym | | p>0.9999 | |
|  | NC vs. Am | | P=0.0003 | | Ah vs. Am | | p>0.9999 | |
|  | Yh vs. Ah | | p>0.9999 | | Ym vs. Am | | p>0.9999 | |
| (b) IL-1β (pg/mg)  F=6.439, p=0.0011 | NC vs. Yh | | P=0.1505 | | Yh vs. Ym | | p>0.9999 | |
|  | NC vs. Ah | | P=0.0006 | | Yh vs. Am | | p>0.9999 | |
|  | NC vs. Ym | | P=0.0496 | | Ah vs. Ym | | P=0.9404 | |
|  | NC vs. Am | | P=0.0102 | | Ah vs. Am | | p>0.9999 | |
|  | Yh vs. Ah | | P=0.3637 | | Ym vs. Am | | p>0.9999 | |
| (C) | | | | | | | | |
|  | | U statistic | | Sum of ranks | | | | P value |
|  |  |  |  | NC | | PH | |  |
| (a) Spont. Alteration (%) | | 0 | | 57 | | 21 | | P=0.0022 |
| (b) IL-1β (pg/mg) | | 0 | | 21 | | 57 | | P=0.0022 |
| (D) | | | | | | | | |
|  | | U statistic | | Sum of ranks | | | | P value |
|  |  |  |  | NC | | PH | |  |
| (a) MPO activity (μunit/mg) | | 0 | | 21 | | 57 | | P=0.0022 |
| (b) IL-1β (pg/mg) | | 0 | | 22 | | 56 | | P=0.0043 |

| Supplemental Figure S5. Figure S2 intensity  two tailed Mann-Whitney U test. | | | | | |
| --- | --- | --- | --- | --- | --- |
|  | | U statics | Sum of ranks | | P value |
|  |  |  | YF | AF |  |
| (A) Western blot | BDNF/β-actin | 0 | 26 | 10 | P=0.0286 |
|  | p-p65/p-65 | 0 | 10 | 26 | P=0.0286 |
| (B) BDNF fluorescent intensity (%) | | 0 | 57 | 21 | P=0.0022 |
| (C) NFκB fluorescent intensity (%) | | 0 | 21 | 57 | P=0.0022 |
| (D) LPS fluorescent intensity (%) | | 0 | 21 | 57 | P=0.0022 |
| (E) IL-1R fluorescent intensity (%) | | 0 | 21 | 57 | P=0.0022 |
| (F) NFκB +CD11c+ cell counting | | 0 | 21 | 57 | P=0.0022 |

| Supplemental Figure S6. PH, EC dose-dependent  one-way ANOVA with post-hoc Holm-Sidak's multiple comparisons test. | | | | |
| --- | --- | --- | --- | --- |
| (A) Spont. Alteration (%)  F=25.06, p<0.0001 | NC vs. PH7 | P=0.0014 | PH7 vs. PH8 | P=0.1954 |
|  | NC vs. PH8 | p<0.0001 | PH7 vs. PH9 | P=0.0014 |
|  | NC vs. PH9 | p<0.0001 | PH8 vs. PH9 | P=0.0193 |
| (B) Spont. Alteration (%)  F=4.409, p=0.0155 | NC vs. EC7 | P=0.3618 | EC7 vs. EC8 | P=0.6638 |
|  | NC vs. EC8 | P=0.4882 | EC7 vs. EC9 | P=0.2126 |
|  | NC vs. EC9 | P=0.0119 | EC8 vs. EC9 | P=0.1121 |

| Supplemental Figure D7. Figure 3 intensity  one-way ANOVA with post-hoc Bonferroni's multiple comparisons test. | | | | |
| --- | --- | --- | --- | --- |
|  | | NC vs. EC | NC vs. PH | EC vs. PH |
| (A) Western blot | BDNF/β-actin  F=33.93, p<0.0001 | p<0.0001 | p<0.0001 | P=0.3207 |
|  | p-p65/p-65  F= 31.95, p <0.0001 | p<0.0001 | P=0.0002 | P=0.0779 |
| (B) BDNF fluorescent intensity (%)  F=470.9, p<0.0001 | | p<0.0001 | p<0.0001 | P=0.8520 |
| (C) NFκB fluorescent intensity (%)  F=65.98, p<0.0001 | | P=0.0005 | p<0.0001 | p<0.0001 |
| (D) TLR4 fluorescent intensity (%)  F=231.8, p<0.0001 | | p<0.0001 | p<0.0001 | p<0.0001 |
| (E) LPS fluorescent intensity (%)  F=220.1, p<0.0001 | | p<0.0001 | P=0.0276 | p<0.0001 |
| (F) IL-1R fluorescent intensity (%)  F=90.40, p<0.0001 | | p<0.0001 | p<0.0001 | p>0.9999 |
| (G) NFκB +CD11c cell counting  F=59.41, p<0.0001 | | p<0.0001 | p<0.0001 | p>0.9999 |

| Supplemental Figure S8. Vagotomy  two tailed Mann-Whitney U test. | | | | |
| --- | --- | --- | --- | --- |
|  | U statics | Sum of ranks | | P value |
|  |  | NC | Vx |  |
| (B) Fecal length (mm) | 1 | 56 | 22 | P=0.0043 |
| (C) Number of feces in 5 mins | 1 | 56 | 22 | P=0.0065 |

| Supplemental Figure S9. Figure 5 intensity  one-way ANOVA with post-hoc Bonferroni's multiple comparisons test. | | | | | |
| --- | --- | --- | --- | --- | --- |
| (A) Western blot | BDNF/β-actin  F= 25.35  p <0.0001 | NC vs. Vx | P=0.2240 | Vx vs. PH | p<0.0001 |
|  |  | NC vs. PH | p<0.0001 | Vx vs. VxPH | P=0.0993 |
|  |  | NC vs. VxPH | p>0.9999 | PH vs. VxPH | p<0.0001 |
| (B) BDNF fluorescent intensity (%)  F=71.06, p<0.0001 | | NC vs. Vx | p>0.9999 | Vx vs. PH | p<0.0001 |
|  |  | NC vs. PH | p<0.0001 | Vx vs. VxPH | P=0.0021 |
|  |  | NC vs. VxPH | P=0.0008 | PH vs. VxPH | p<0.0001 |
| (C) NFκB fluorescent intensity (%)  F=25.25, p<0.0001 | | NC vs. Vx | p>0.9999 | Vx vs. PH | p<0.0001 |
|  |  | NC vs. PH | p<0.0001 | Vx vs. VxPH | p>0.9999 |
|  |  | NC vs. VxPH | p>0.9999 | PH vs. VxPH | p<0.0001 |
| (D) LPS fluorescent intensity (%)  F=26.51, p<0.0001 | | NC vs. Vx | p>0.9999 | Vx vs. PH | p<0.0001 |
|  |  | NC vs. PH | p<0.0001 | Vx vs. VxPH | p<0.0001 |
|  |  | NC vs. VxPH | p<0.0001 | PH vs. VxPH | p>0.9999 |
| (E) IL-1R fluorescent intensity (%)  F=90.81, p<0.0001 | | NC vs. Vx | p>0.9999 | Vx vs. PH | p<0.0001 |
|  |  | NC vs. PH | p<0.0001 | Vx vs. VxPH | P=0.0128 |
|  |  | NC vs. VxPH | P=0.0068 | PH vs. VxPH | p<0.0001 |
| (F) NFκB + CD11c+ cell counting  F=108.9, p<0.0001 | | NC vs. Vx | p>0.9999 | Vx vs. PH | p<0.0001 |
|  |  | NC vs. PH | p<0.0001 | Vx vs. VxPH | p<0.0001 |
|  |  | NC vs. VxPH | p<0.0001 | PH vs. VxPH | p>0.9999 |

| Supplemental Figure S11. Figure 6 intensity  one-way ANOVA with post-hoc Bonferroni's multiple comparisons test. | | | | | |
| --- | --- | --- | --- | --- | --- |
| (A) Western blot | BDNF/β-actin  F=26.98, p<0.0001 | NC vs. Vx | p>0.9999 | Vx vs. EC | p<0.0001 |
|  |  | NC vs. EC | p<0.0001 | Vx vs. VxEC | P=0.0001 |
|  |  | NC vs. VxEC | p<0.0001 | EC vs. VxEC | p>0.9999 |
| (B) BDNF fluorescent intensity (%)  F=66.20, p<0.0001 | | NC vs. Vx | P=0.5132 | Vx vs. PH | p<0.0001 |
|  |  | NC vs. PH | p<0.0001 | Vx vs. VxPH | p<0.0001 |
|  |  | NC vs. VxPH | p<0.0001 | PH vs. VxPH | p>0.9999 |
| (C) NFκB fluorescent intensity (%)  F=11.53, p=0.0001 | | NC vs. Vx | p>0.9999 | Vx vs. PH | P=0.0048 |
|  |  | NC vs. PH | P=0.0015 | Vx vs. VxPH | P=0.0061 |
|  |  | NC vs. VxPH | P=0.0019 | PH vs. VxPH | p>0.9999 |
| (D) LPS fluorescent intensity (%)  F=57.01, p<0.0001 | | NC vs. Vx | p>0.9999 | Vx vs. PH | p<0.0001 |
|  |  | NC vs. PH | p<0.0001 | Vx vs. VxPH | p<0.0001 |
|  |  | NC vs. VxPH | p<0.0001 | PH vs. VxPH | p>0.9999 |
| (E) IL-1R fluorescent intensity (%)  F=225.7, p<0.0001 | | NC vs. Vx | p>0.9999 | Vx vs. PH | p<0.0001 |
|  |  | NC vs. PH | p<0.0001 | Vx vs. VxPH | p<0.0001 |
|  |  | NC vs. VxPH | p<0.0001 | PH vs. VxPH | p>0.9999 |
| (F) NFκB +CD11c cell counting  F=90.47, p<0.0001 | | NC vs. Vx | p>0.9999 | Vx vs. PH | p<0.0001 |
|  |  | NC vs. PH | p<0.0001 | Vx vs. VxPH | p<0.0001 |
|  |  | NC vs. VxPH | p<0.0001 | PH vs. VxPH | p>0.9999 |

| Supplemental Figure S12.  one-way ANOVA with post-hoc Bonferroni's multiple comparisons test | | | | |
| --- | --- | --- | --- | --- |
| (A) Spont. alteration (%)  F=10.46, p<0.0001 | NC vs. LPS | P=0.0012 | LPS vs. EV+LPS | p>0.9999 |
|  | NC vs. EV | P=0.0006 | LPS vs. LPSip | p>0.9999 |
|  | NC vs. EV+LPS | p<0.0001 | EV vs. EV+LPS | p>0.9999 |
|  | NC vs. LPSip | P=0.0111 | EV vs. LPSip | p>0.9999 |
|  | LPS vs. EV | p>0.9999 | EV+LPS vs. LPSip | P=0.2800 |
| (F) IL-1β (pg/mg)  F=13.33, p<0.0001 | NC vs. LPS | p<0.0001 | LPS vs. EV+LPS | p>0.9999 |
|  | NC vs. EV | p<0.0001 | LPS vs. LPSip | p>0.9999 |
|  | NC vs. EV+LPS | p<0.0001 | EV vs. EV+LPS | p>0.9999 |
|  | NC vs. LPSip | p<0.0001 | EV vs. LPSip | p>0.9999 |
|  | LPS vs. EV | p>0.9999 | EV+LPS vs. LPSip | p>0.9999 |
| (G) LPS (ng/mL)  F=32.59, p<0.0001 | NC vs. LPS | p<0.0001 | LPS vs. EV+LPS | p>0.9999 |
|  | NC vs. EV | P=0.0254 | LPS vs. LPSip | P=0.0123 |
|  | NC vs. EV+LPS | p<0.0001 | EV vs. EV+LPS | P=0.0087 |
|  | NC vs. LPSip | p<0.0001 | EV vs. LPSip | p<0.0001 |
|  | LPS vs. EV | P=0.0148 | EV+LPS vs. LPSip | P=0.0208 |
| (H) Colon length (cm)  F=  F=17.42, p<0.0001 | NC vs. LPS | P=0.0099 | LPS vs. EV+LPS | P=0.2978 |
|  | NC vs. EV | P=0.1428 | LPS vs. LPSip | P=0.0074 |
|  | NC vs. EV+LPS | p<0.0001 | EV vs. EV+LPS | P=0.0226 |
|  | NC vs. LPSip | p<0.0001 | EV vs. LPSip | P=0.0004 |
|  | LPS vs. EV | p>0.9999 | EV+LPS vs. LPSip | p>0.9999 |
| (I) MPO activity (μunit/mg)  F= 19.22, p <0.0001 | NC vs. LPS | P=0.0004 | LPS vs. EV+LPS | P=0.6983 |
|  | NC vs. EV | P=0.1120 | LPS vs. LPSip | P=0.1407 |
|  | NC vs. EV+LPS | p<0.0001 | EV vs. EV+LPS | P=0.0039 |
|  | NC vs. LPSip | p<0.0001 | EV vs. LPSip | P=0.0006 |
|  | LPS vs. EV | P=0.3723 | EV+LPS vs. LPSip | p>0.9999 |
| (J) IL-1β (pg/mg)  F=8.019, p<0.0003 | NC vs. LPS | P=0.0227 | LPS vs. EV+LPS | p>0.9999 |
|  | NC vs. EV | P=0.0131 | LPS vs. LPSip | P=0.4280 |
|  | NC vs. EV+LPS | P=0.0147 | EV vs. EV+LPS | p>0.9999 |
|  | NC vs. LPSip | p<0.0001 | EV vs. LPSip | P=0.6713 |
|  | LPS vs. EV | p>0.9999 | EV+LPS vs. LPSip | P=0.6099 |
| (L) LPS (ng/mg)  F=52.34, p<0.0001 | NC vs. LPS | p<0.0001 | LPS vs. EV+LPS | P=0.1647 |
|  | NC vs. EV | P=0.0029 | LPS vs. LPSip | P=0.0005 |
|  | NC vs. EV+LPS | p<0.0001 | EV vs. EV+LPS | p<0.0001 |
|  | NC vs. LPSip | p<0.0001 | EV vs. LPSip | p<0.0001 |
|  | LPS vs. EV | P=0.0092 | EV+LPS vs. LPSip | P=0.2785 |

| Supplemental Figure S13. Figure 7 intensity  one-way ANOVA with post-hoc Bonferroni's multiple comparisons test. | | | | | |
| --- | --- | --- | --- | --- | --- |
| (A)  Western blot | BDNF/β-actin  F=219.5, p<0.0001 | NC vs. Vx | p>0.9999 | Vx vs. VxLPS | p<0.0001 |
|  |  | NC vs. EV | p<0.0001 | EV vs. VxEV | p<0.0001 |
|  |  | NC vs. VxEV | p<0.0001 | EV vs. LPS | p>0.9999 |
|  |  | NC vs. LPS | p<0.0001 | EV vs. VxLPS | p>0.9999 |
|  |  | NC vs. VxLPS | p<0.0001 | VxEV vs. LPS | p<0.0001 |
|  |  | Vx vs. EV | p<0.0001 | VxEV vs. VxLPS | p<0.0001 |
|  |  | Vx vs. VxEV | p<0.0001 | LPS vs. VxLPS | p>0.9999 |
|  |  | Vx vs. LPS | p<0.0001 |  |  |
| (B) BDNF fluorescent intensity (%)  F=150.4, p<0.0001 | | NC vs. Vx | P=0.9854 | Vx vs. VxLPS | p<0.0001 |
|  |  | NC vs. EV | p<0.0001 | EV vs. VxEV | p<0.0001 |
|  |  | NC vs. VxEV | p<0.0001 | EV vs. LPS | P=0.6613 |
|  |  | NC vs. LPS | p<0.0001 | EV vs. VxLPS | P=0.6477 |
|  |  | NC vs. VxLPS | p<0.0001 | VxEV vs. LPS | p<0.0001 |
|  |  | Vx vs. EV | p<0.0001 | VxEV vs. VxLPS | p<0.0001 |
|  |  | Vx vs. VxEV | p<0.0001 | LPS vs. VxLPS | p>0.9999 |
|  |  | Vx vs. LPS | p<0.0001 |  |  |
| (C) NFκB fluorescent intensity (%)  F=204.3, p<0.0001 | | NC vs. Vx | p>0.9999 | Vx vs. VxLPS | p<0.0001 |
|  |  | NC vs. EV | p<0.0001 | EV vs. VxEV | p<0.0001 |
|  |  | NC vs. VxEV | P=0.0008 | EV vs. LPS | p>0.9999 |
|  |  | NC vs. LPS | p<0.0001 | EV vs. VxLPS | p>0.9999 |
|  |  | NC vs. VxLPS | p<0.0001 | VxEV vs. LPS | p<0.0001 |
|  |  | Vx vs. EV | p<0.0001 | VxEV vs. VxLPS | p<0.0001 |
|  |  | Vx vs. VxEV | P=0.0080 | LPS vs. VxLPS | p>0.9999 |
|  |  | Vx vs. LPS | p<0.0001 |  | p<0.0001 |
| (D) LPS fluorescent intensity (%)  F=20.86, p<0.0001 | | NC vs. Vx | p>0.9999 | Vx vs. VxLPS | p<0.0001 |
|  |  | NC vs. EV | P=0.0004 | EV vs. VxEV | p>0.9999 |
|  |  | NC vs. VxEV | P=0.0069 | EV vs. LPS | P=0.7059 |
|  |  | NC vs. LPS | p<0.0001 | EV vs. VxLPS | P=0.3547 |
|  |  | NC vs. VxLPS | p<0.0001 | VxEV vs. LPS | P=0.0712 |
|  |  | Vx vs. EV | P=0.0005 | VxEV vs. VxLPS | P=0.0318 |
|  |  | Vx vs. VxEV | P=0.0077 | LPS vs. VxLPS | p>0.9999 |
|  |  | Vx vs. LPS | p<0.0001 |  |  |
| (E) IL-1R fluorescent intensity (%)  F=48.88, p<0.0001 | | NC vs. Vx | p>0.9999 | Vx vs. VxLPS | p<0.0001 |
|  |  | NC vs. EV | p<0.0001 | EV vs. VxEV | p<0.0001 |
|  |  | NC vs. VxEV | P=0.0275 | EV vs. LPS | p>0.9999 |
|  |  | NC vs. LPS | p<0.0001 | EV vs. VxLPS | p>0.9999 |
|  |  | NC vs. VxLPS | p<0.0001 | VxEV vs. LPS | p<0.0001 |
|  |  | Vx vs. EV | p<0.0001 | VxEV vs. VxLPS | p<0.0001 |
|  |  | Vx vs. VxEV | P=0.5137 | LPS vs. VxLPS | p>0.9999 |
|  |  | Vx vs. LPS | p<0.0001 |  |  |
| (F) NFκB + CD11c+ cell counting  F=74.10, p<0.0001 | | NC vs. Vx | p>0.9999 | Vx vs. VxLPS | p<0.0001 |
|  |  | NC vs. EV | P=0.0284 | EV vs. VxEV | p>0.9999 |
|  |  | NC vs. VxEV | P=0.0126 | EV vs. LPS | p<0.0001 |
|  |  | NC vs. LPS | p<0.0001 | EV vs. VxLPS | p<0.0001 |
|  |  | NC vs. VxLPS | p<0.0001 | VxEV vs. LPS | p<0.0001 |
|  |  | Vx vs. EV | P=0.0376 | VxEV vs. VxLPS | p<0.0001 |
|  |  | Vx vs. VxEV | P=0.0168 | LPS vs. VxLPS | p>0.9999 |
|  |  | Vx vs. LPS | p<0.0001 |  |  |

| Supplemental Figure S14. Figure 8A intensity  one-way ANOVA with post-hoc Bonferroni's multiple comparisons test. | | | | |
| --- | --- | --- | --- | --- |
| FITC-fluorescent intensity (%) | NC vs. Vx | p>0.9999 | Vx vs. VsLPS | p<0.0001 |
|  | NC vs. EV | p<0.0001 | EV vs. VxEV | p<0.0001 |
|  | NC vs. VxEV | p<0.0001 | EV vs. LPS | p<0.0001 |
|  | NC vs. LPS | p<0.0001 | EV vs. VsLPS | p<0.0001 |
|  | NC vs. VsLPS | p<0.0001 | VxEV vs. LPS | p>0.9999 |
|  | Vx vs. EV | p<0.0001 | VxEV vs. VsLPS | p>0.9999 |
|  | Vx vs. VxEV | p<0.0001 | LPS vs. VsLPS | p>0.9999 |

| Supplemental Figure S15. GABA and NMDA receptors  one-way ANOVA with post-hoc Bonferroni's multiple comparisons test. | | | |
| --- | --- | --- | --- |
|  | NC vs. EC | NC vs. PH | YF vs. AF |
| GABA_Aα2_ (fold change) | p>0.9999 | P=0.0277 | P=0.1431 |
| GABA_B1b_ (fold change) | p>0.9999 | P=0.0140 | P=0.0319 |
| GABA_Aα1_ (fold change) | P=0.4696 | P=>0.9999 | P=0.4130 |
| NMDA (fold change) | p>0.9999 | P=>0.9999 | p>0.9999 |

| Supplemental Figure S19.  one-way ANOVA with post-hoc Bonferroni's multiple comparisons test. | | | | |
| --- | --- | --- | --- | --- |
| Spont. alteration (%) | NC vs. Vx | p>0.9999 | VxPH vs. VxEV | p>0.9999 |
|  | NC vs. PH | p<0.0001 | VxPH vs. LPS | p>0.9999 |
|  | NC vs. VxPH | P=0.0027 | VxPH vs. VxLPS | p>0.9999 |
|  | NC vs. EC | p<0.0001 | VxPH vs. EV+LPS | P=0.0414 |
|  | NC vs. VxEC | p<0.0001 | VxPH vs. LPSip | p>0.9999 |
|  | NC vs. EV | p<0.0001 | EC vs. VxEC | p>0.9999 |
|  | NC vs. VxEV | P=0.0480 | EC vs. EV | p>0.9999 |
|  | NC vs. LPS | p<0.0001 | EC vs. VxEV | p>0.9999 |
|  | NC vs. VxLPS | p<0.0001 | EC vs. LPS | p>0.9999 |
|  | NC vs. EV+LPS | p<0.0001 | EC vs. VxLPS | P>0.9999 |
|  | NC vs. LPSip | P=0.0039 | EC vs. EV+LPS | p>0.9999 |
|  | Vx vs. PH | p<0.0001 | EC vs. LPSip | P>0.9999 |
|  | Vx vs. VxPH | P=0.5527 | VxEC vs. EV | P=0.7984 |
|  | Vx vs. EC | p<0.0001 | VxEC vs. VxEV | p>0.9999 |
|  | Vx vs. VxEC | P=0.0028 | VxEC vs. LPS | p>0.9999 |
|  | Vx vs. EV | p<0.0001 | VxEC vs. VxLPS | p>0.9999 |
|  | Vx vs. VxEV | p>0.9999 | VxEC vs. EV+LPS | p>0.9999 |
|  | Vx vs. LPS | P=0.0007 | VxEC vs. LPSip | p>0.9999 |
|  | Vx vs. VxLPS | P=0.0059 | EV vs. VxEV | P=0.0109 |
|  | Vx vs. EV+LPS | p<0.0001 | EV vs. LPS | p>0.9999 |
|  | Vx vs. LPSip | P=0.2354 | EV vs. VxLPS | p>0.9999 |
|  | PH vs. VxPH | P=0.0339 | EV vs. EV+LPS | p>0.9999 |
|  | PH vs. EC | p>0.9999 | EV vs. LPSip | p>0.9999 |
|  | PH vs. VxEC | p>0.9999 | VxEV vs. LPS | p>0.9999 |
|  | PH vs. EV | p>0.9999 | VxEV vs. VxLPS | p>0.9999 |
|  | PH vs. VxEV | P=0.1219 | VxEV vs. EV+LPS | P=0.1233 |
|  | PH vs. LPS | p>0.9999 | VxEV vs. LPSip | p>0.9999 |
|  | PH vs. VxLPS | p>0.9999 | LPS vs. VxLPS | p>0.9999 |
|  | PH vs. EV+LPS | p>0.9999 | LPS vs. EV+LPS | p>0.9999 |
|  | PH vs. LPSip | p>0.9999 | LPS vs. LPSip | p>0.9999 |
|  | VxPH vs. EC | p>0.9999 | VxLPS vs. EV+LPS | p>0.9999 |
|  | VxPH vs. VxEC | p>0.9999 | VxLPS vs. LPSip | p>0.9999 |
|  | VxPH vs. EV | P=0.0013 | EV+LPS vs. LPSip | p>0.9999 |

**[Methods]**

**Mouse breeding and gut bacteria *Paenalcaligenes hominis* and *Escherichia coli* assay**

To understand whether infected gut bacteria *Paenalcaligenes hominis* and *Escherichia coli* were transmitted from parents to offspring, *Paenalcaligenes hominis* or *Escherichia coli* (1×10^9^ CFU/day/mouse) were orally gavaged in SPF male and female mice (8 weeks old) daily for 8 days. Control mice were treated with saline instead of the gut bacterial suspension. Female mice treated with or without gut bacteria were housed 3:1 with male mice treated with or without gut bacteria, respectively, 6 h after the final gavage of gut bacteria. Male mice were removed from the cages with female mice after 5 consecutive nights. Each pregnant dam was single-housed. Offspring feces were collected on the postnatal day 14 and the populations of *Paenalcaligenes hominis* or *Escherichia coli* were assayed by qPCR.

**Purification of LPS from *Paenalcaligenes hominis* (PH)**

PH was cultures in tryptic soy broth Briefly, PH was cultured in tryptic soy broth (500 mL) for 24 h at 37°C and collected by centrifugation at 10 000 g for 5 min according to Jang et al. [1]. The pellets were washed twice in 0.15 M phosphate-buffered saline (PBS, pH 7.2) containing 0.15 mM CaCl_2_ and 0.5 mM MgCl_2_, suspended in 10 mL PBS, and sonicated for 30 min on ice. The sonicate was incubated with proteinase K (100 μg/mL, Sigma, St Louis, MO) at 65°C for 1 h and subsequently treated with RNase (40 μg/mL, Sigma) and DNase (20 μg/mL, Sigma) in the presence of 1 μL/mL of 20% MgSO_4_ and 4 μL/mL of chloroform at 37°C overnight. The reaction solution was extracted with the same volume of 90% phenol with vigorous shaking at 65 – 70°C for 15 min, transferred to polypropylene tubes, and centrifuged at 8,500 g for 15 min. The supernatants were treated with 10 volumes of 95% cold ethanol in the presence of 0.5 M sodium acetate at −20°C overnight and centrifuged at 2,000 g at 4°C for 10 min. The resulting pellet was suspended in distilled water, dialyzed twice against double distilled water at 4°C, then lyophilized, and used in the present experiment as LPS.

**Properties of extracellular vesicles purified from *Paennalcaligenes hominis* (PH)**

*Paennalcaligenes hominis* EV consisted of proteins, LPS, and nucleic acid. The ratio of protein, LPS, and nucleic acid was approximately 10 μg/32 ng/15 ng. The average EV size was 0.43 ± 0.02 μm.

**Quantitative real time – polymerase chain reaction (qPCR) for GABA receptors**

qPCRs for GABA_A_α1, GABA_A_α2, GABA_B_1b, and N-methyl-D-aspartate (NMDA) receptors and GAPDH were performed on the Rotor-Gene Q® using DNA polymerase and SYBR Green I (a reaction volume, 20 μL), as previously reported [2]. Primers for qPCR are described in Supplement Table S4 [3-6]. The normalized expression of each target gene, as for GAPDH, was calculated for all samples using Microsoft Excel.

**References**

1. Jang SE, Lim SM, Jeong JJ, Jang HM, Lee HJ, Han MJ, Kim DH. Gastrointestinal inflammation by gut microbiota disturbance induces memory impairment in mice. Mucosal Immunol. 2018;11: 369-379.

2. Jang SE, Jeong JJ, Kim JK, Han MJ, Kim DH. Simultaneous Amelioratation of Colitis and Liver Injury in Mice by Bifidobacterium longum LC67 and Lactobacillus plantarum LC27. Sci. Rep. 2018;8:7500.

3. Kanold PO, Kim YA, GrandPre T, Shatz CJ. Co-regulation of ocular dominance plasticity and NMDA receptor subunit expression in glutamic acid decarboxylase-65 knock-out mice. J. Physiol. 2009;587(Pt 12): 2857-2867.

4. Tan S, Rudd JA, Yew DT. Gene expression changes in GABA(A) receptors and cognition following chronic ketamine administration in mice. PLoS One 2011;6: e21328.

5. Kang JY, Chadchankar J, Vien TN, Mighdoll MI, Hyde TM, Mather RJ, Deeb TZ, Pangalos MN, Brandon NJ, Dunlop J, Moss SJ. Deficits in the activity of presynaptic γ-aminobutyric acid type B receptors contribute to altered neuronal excitability in fragile X syndrome. J. Biol. Chem. 2017;292: 6621-6632.

6. Ze X, Su M, Zhao X, Jiang H, Hong J, Yu X, Liu D, Xu B, Sheng L, Zhou Q, Zhou J, Cui J, Li K, Wang L, Ze Y, Hong F. TiO2 nanoparticle-induced neurotoxicity may be involved in dysfunction of glutamate metabolism and its receptor expression in mice. Environ. Toxicol. 2016;31: 655-662.
